# CORE-Kids: a protocol for the development of a core outcome set for childhood fractures

**DOI:** 10.1136/bmjopen-2019-036224

**Published:** 2020-02-28

**Authors:** Ben Arthur Marson, Joseph C Manning, Marilyn James, Simon Craxford, Sandeep R Deshmukh, Benjamin J Ollivere, The CORE-Kids Group

**Affiliations:** 1 Trauma Outcomes Group, University of Nottingham, Nottingham, UK; 2 School of Health Sciences, University of Nottingham, Nottingham, UK; 3 School of Medicine, University of Nottingham, Nottingham, UK

**Keywords:** paediatric orthopaedics, trauma management, qualitative research

## Abstract

**Introduction:**

Limb fractures in children are common yet there are few trials that compare treatments for these injuries. There is significant heterogeneity in the outcomes reported in the paediatric orthopaedic literature, which limits the ability to compare study results and draw firm conclusions. The aim of the CORE-Kids Study is to develop a core outcome set for use in research studies of childhood limb fractures. A core outcome set will provide a minimum set of outcomes to be measured in all trials to minimise the heterogeneity of outcomes reported and minimise reporting bias. A core outcome set ensures that outcomes are reported that are relevant to families as well as clinicians. The core outcome set will include additional upper and lower limb modules.

**Methods:**

The development of the core outcome set will require four phases to evaluate:

1. What are the outcomes that are relevant to professionals?
2. What are the outcomes that are relevant to families?
3. What are the most important of these outcomes?
4. Which outcomes should be included in the core outcome set?

This will be completed through a systematic review of trials to identify the outcomes domains that are relevant to trialists. A series of semi-structured interviews will be completed with families to identify the outcome domains that are relevant to families. These outcome domains will be used in a three-round Delphi Study to analyse the importance of these outcome domains to a range of stakeholders including parents, clinicians and researchers. Following this, the core outcome set will be decided at a consensus meeting.

**Ethics and dissemination:**

Ethical approval has been awarded HRA/REC IRAS number 262503. Date of approval 06/08/2019. Dissemination will be through scientific literature and international societies.

**Trial registration:**

Core Outcome Measures in Effectiveness Trials Initiative, registration number: 1274. Date of registration 13/12/2018.

**PROSPERO registration number:**

CRD42018106605.

**Strengths and limitations of this study**

- This is a core outcome set with a broad scope that will be widely applicable to children’s fractures and injuries.
- The core outcome set draws from experience from a wide range of stakeholders to ensure that the outcomes selected are relevant.
- The scope of this study is to deliver an outcome set for children aged 5–15 for randomised trials. It may be applicable to all research studies and to infants and adolescents, but further work will be required to confirm this.

## Introduction

The WHO and UNICEF have identified childhood injury as a leading cause of mortality and morbidity in low and middle income countries.^1^ The precise global burden of childhood fractures is not known, but 1 in 3 children in Europe will sustain a fracture by their 17th birthday.^2^

There are six Cochrane reviews that have evaluated the literature supporting the management of childhood fractures.^3–8^ The reviews have identified 45 trials with sufficient quality to inform practice, with most trials relating to the management of wrist fractures. Only two reviews were able to draw firm conclusions due to a paucity of randomised trials in children’s fracture care, despite the relative high frequency of these injuries.^5 7^

There is a need to deliver high quality randomised trials to evaluate treatments in childhood fractures, and these trials need to be underpinned by a robust rationale for the selection of primary and secondary outcomes.^9–11^ There is a growing use of patient reported outcomes in paediatric orthopaedics with little agreement regarding the selection of outcome tools to compare treatments.^12 13^

To compare treatments or interventions, an outcome needs to be identified which encompasses a feature of the condition to measure and a technique to measure it. An outcome domain is the broad feature of the condition or a patient’s health that is being measured (eg, pain, range of movement or function), whereas the outcome tool is the technique used to measure it.^14^

The heterogeneity of reporting of outcomes can be improved through the development and adoption of a core outcome set.^15 16^ This is an agreed minimum standard group of outcomes that are reported in every research study.^17^ Through using a core set and adding any additional relevant secondary outcomes, trialists can ensure that their results are relevant to surgeons, families and other stakeholders and can be effectively combined for evidence synthesis and guideline generation.^14^

The Core Outcome Measures in Effectiveness Trials (COMET) Initiative is an international collaboration with the objective of promoting the development and use of core outcome sets. The COMET Initiative has published a handbook evaluating the available methodology for developing a core outcome set^18^ and reporting standards for the development and reporting of core outcome sets.^19 20^

There are only a few core outcome sets that have been developed for use in orthopaedic trials.^21^ For trials relating to fractures, the only core outcome set available has been developed for trials of hip fractures in the elderly which would not be appropriate to extrapolate onto fractures in children.^22^ A core outcome set is in development for medial epicondyle fractures in children, but this is a relatively uncommon injury and is focussed on a single fracture type.^23^

The CORE-Kids Study will develop a core outcome set identifying the core domains that should be measured in every trial of childhood fractures. This protocol has been devised in line with the Core Outcome Set- STAndard Protocol Items (COS-STAP) statement^24^ and the development studies will be completed and reported as per the Core Outcome Set- Standadards for development (COS-STAD) and Core Outcome Set- Standadards for reporting (COS-STAR) statements.^19 20^

### Study scope

The scope for this core outcome set has been set by the steering group in conjunction with the National Institute for Health Research Trauma Trials network.

The scope for this core outcome set is:

- Setting: research studies.
- Health condition: fractures to the appendicular skeleton (ie, limbs, pelvis, shoulder girdle but not spine, ribs or head) excluding children with multiple injuries. The core outcome set will be divided into three modules: a central ‘all fractures’ set, an ‘upper limb’ set and a ‘lower limb’ set.
- Target population: school aged children (aged 5–16)—it is anticipated that infants (0–4) and older adolescents (17–18) may share common core outcomes but this cannot be assumed and should be confirmed in future work.
- Target interventions: treatment for fractures, both involving surgical and non-surgical (conservative) techniques.

## Methods and design

The development of the core outcome set will require four phases to evaluate:

- What are the outcomes that are relevant to trialists?
- What are the outcomes that are relevant to families?
- What are the most important of these outcomes?
- Which outcomes should be included in the core outcome set?

These questions will be answered sequentially through a systematic review of trials, interviews with families, an international online Delphi Study and a consensus meeting.

### What are the outcomes that are relevant to professionals?

In order to understand the outcomes most relevant to professionals, a systematic review of all trials in childhood fractures will be completed. This systematic review will identify all outcomes that have been reported in trials relating to childhood fractures. The review has been prospectively registered in the PROSPERO database^25^ and will be reported according to Preferred Reporting Items for Systematic Reviews and Meta-Analyses statement.^26^

Studies that are randomised or quasi-randomised will be included where the majority of the participants are children (age <16) who have sustained fractures to the appendicular skeleton or dislocations to large joints. All interventions that are designed to treat fractures will be considered, with the exclusion of studies that evaluate the efficacy of anaesthetic or analgesia techniques in isolation. Study protocols and trial registrations of randomised or quasi-randomised trials will also be included where outcomes are identified.

English language interventional trials of childhood fractures will be identified by searching electronic database, reviewing bibliographies of Cochrane reviews and hand searching of trial registries and online search engines. The electronic databases that will be searched are OVID MEDLINE, OVID Embase and the Cochrane Central Register of Controlled Trial. A manual search of clinicaltrials.gov, the International Standard Randomised Controlled Trial Number (ISRCTN) registry and WHO International Clinical Trials Registry Platform (ICTRP) will be completed to identify any unpublished or in-progress trials.

Titles will be screened by one researcher and full texts analysed by two researchers. Outcomes and outcome tools will be extracted and classified according to the WHO International classification of functioning (WHO ICF).^27^

No formal assessment of study quality or risk of bias will be completed as the objective of this review is to identify outcomes reported in trials rather than prioritise outcomes or evaluate treatment options. Therefore, the quality of each study will not add any additional value to this review. This is consistent with other systematic review of outcomes reported in different medical and surgical conditions.^28–30^

### What are the outcomes that are relevant to families?

The outcomes that are relevant to families will be evaluated through a series of semi-structured interviews with parent–child dyads (composed of the child and at least one parent or carer) where the child has recent experience of breaking a bone in the appendicular skeleton. The use of semi-structured interview design has been selected to maximise the depth of the data that will be obtained while ensuring that different domains within the WHO ICF are covered. Interviews will be completed in the fracture clinic as a familiar environment following the child’s final clinical review.^31 32^

Interviews will be conducted by a PhD research student with supervision from an expert in children’s qualitative research. The PhD student will receive training in interview skills and analysis techniques prior to completing these aspects of the study.

The sample size has been set to achieve data saturation. A target of 20 interviews will be conducted with parent–child dyads. Parent–child dyads have been selected to provide a familiar adult for the child, to allow the child to express their opinions and obtain the combined experience of parents and children.^31 33 34^ Sample size has been determined as a minimum required to achieve saturation after consideration of the scope of identifying outcomes and that the nature of the topic involves discussing issues that are not sensitive or distressing.^35^

Heterogeneous, purposeful sampling will be applied to achieve a mix of families with children of different genders, fracture sites (upper and lower limb), fracture severity (inpatient or outpatient treatment) and experience of surgery and experience of cast treatment.^36^ Families will be invited to volunteer and participate through posters in the clinic.

A semi-structured interview schedule has been developed based on the recommendations of Selb *et al*.^37^ The schedule has been modified following pilot testing with a parent involvement group of families. Interviews will be recorded and transcribed.

As advocated in the literature, art-based approaches (draw and tell, graphic elicitation) will be used to facilitate the involvement and verbal responses from child participants.^38 39^ The focus of the semi-structured interviews will be on exploring the following areas: (1) experience of injury; (2) frustrations of recovering and (3) features of recovery and positive and negative outcomes.

A content analysis will be performed to identify the outcome domains that are identified by parents and children that are relevant to them during the experience of injury and recovery. The outcome domains will be classified according to the WHO ICF based on the linking rules by Cieza *et al* to maintain consistency with the outcomes that are relevant to trialists identified during the systematic review.^40 41^ Coding for the content analysis will be performed by two researchers and a saturation table completed using the identified WHO ICF domains to ensure that saturation is achieved prior to completion of the study.

The findings from these interviews will be combined with the results of the systematic review to draw a long list of outcome domains for evaluation using consensus methodology.

### What are the most important of these outcomes?

The long list of candidate outcomes will be reduced through an international three-round Delphi Survey involving relevant stakeholders. As a method of developing consensus, the Delphi technique involves obtaining successive scores for each outcome from each expert panellist following feedback over multiple survey rounds.^42 43^

The Delphi Survey has been designed so that each outcome domain will be evaluated twice: once for importance for upper limb trials and once for lower limb trials. All outcomes will be reviewed by our patent participation group to ensure comprehensibility by lay panellists as well as professionals.

A three-round design has been selected to maximise the chance of delivering consensus, while minimising the attrition bias that the survey is exposed to if too many rounds are undertaken. In the general medical literature and in the developments of core outcome sets, the median and upper quartile number of rounds completed is three rounds,^44 45^ with only 6% of Delphi studies used to agree importance of outcomes incorporating more than three rounds.^46^

As no feedback mechanism has been proven to be most effective in generating consensus,^47^ we will provide multiple-combined feedback from all groups to minimise the number of results that need to be interpreted by panellists.

The Delphi Study will be completed as an online survey using the onlinesurveys.ac.uk interface (JISC, Bristol, UK). Panellists will be assigned a unique code to access the online survey system to permit monitoring of survey completion and attrition. All panellists will be sent up to four email reminders of survey deadlines to maximise completion.

Expert panellists will be approached to represent the following stakeholder groups:

- Parents of children who have had a broken bone.
- Doctors or surgeons who treat children with broken bones.
- Paediatric nurses and therapists.
- Teachers.
- Researchers who have been involved with studies for children with broken bones.
- Systematic reviewers who have been involved with reviews of studies for children with broken bones.

Panellists will be assigned a unique code to access the online survey system to permit recording of survey completion and attrition. All panellists will be sent e-mail reminders of survey deadlines to maximise completion.

For each outcome domain, panellists will assign scores from 1 to 9 (very important) using a Likert Scale. Each outcome domain will be scored twice in the first round, once for upper limb and once for lower limb fractures. In subsequent rounds, outcome domains may be scored once or twice, depending if consensus has been reached.

**Figure 1:**
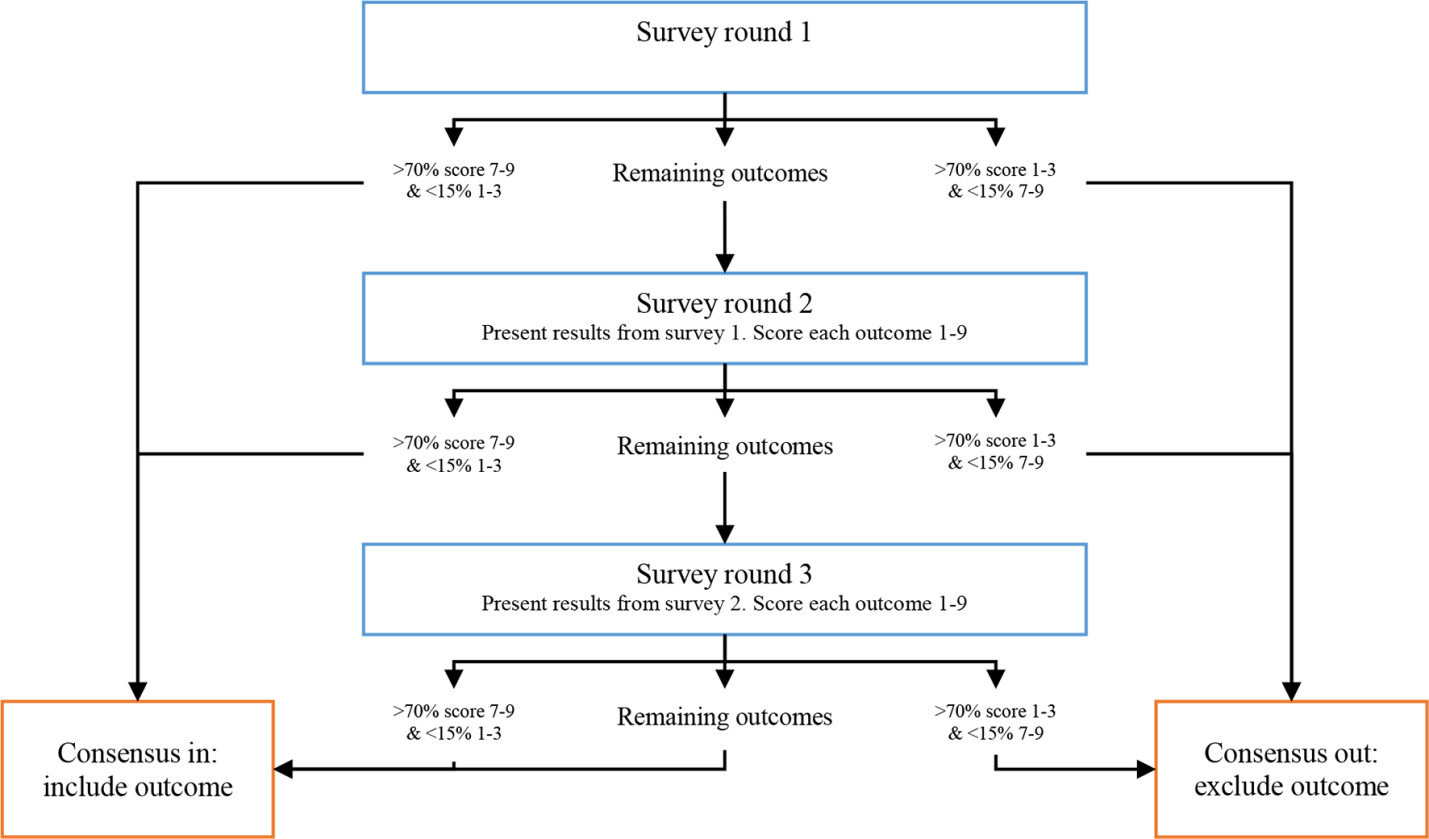
Study flow chart for the three-round Delphi Study. Outcome domains will be identified as ‘consensus in’ if 70% of panellists rate the outcome domain with a score of 7–9 and fewer than 15% of panellists score the domain 1–3. Outcome domains will be identified as ‘consensus out’ if 70% of panellists score the outcome domain 1–3 and fewer than 15% of panellists score the domain 7–9.

In order to minimise the attrition bias associated with Delphi Surveys with higher numbers of items,^46^ outcome domains will be removed from the second and third rounds if consensus is reached. Outcome domains will be removed from further Delphi rounds if consensus in (>70% panellists ranking an outcome with a score of 7–9 and fewer than 15% scoring the outcome 1–3) or consensus out (>70% panellists ranking an outcome with a score of 1–3 and fewer than 15% scoring the outcome 7–9) is achieved (figure 1). This consensus threshold has been selected as it is consistent with previous methodology for the development of core outcome sets^48–50^ and ensures that outcome domains are only retained or exclude if there is a significant majority voting in favour with a small minority expressing the opposite opinion.^17^

No sample size calculations may be performed to guide the panel size for this Delphi. As a target, 75–100 panellists will be recruited to permit for attrition between rounds and to ensure that each stakeholder group has representation.^18^ The stakeholder groups will be pooled into a single panel for completion of the Delphi Survey and in the preparation of feedback between survey rounds. This is to minimise the research burden on panellists and to maximise the likelihood of reaching consensus.^18^ A key potential concern with this methodology is that it is possible for the opinion of minority key stakeholder groups to be overwhelmed if the stakeholder balance is not controlled. To prevent this situation, three key stakeholder groups (1. doctors and researchers; 2. nurses and therapists and 3. parents) will each contribute to a target minimum of 20% of the total participants in the panel.

During the first round of Delphi, panellists will be invited to contribute any additional relevant outcomes that have not been captured in the systematic review or qualitative interviews for inclusion in the second-round survey. Outcome domains that are consensus in or remain following the three rounds of Delphi will be included in the consensus meeting for consideration for the core outcome set.

Additional outcomes that are suggested in the first-round survey will be coded by two researchers to ensure that it represents a new outcome. Where there is disagreement between the two researchers, the senior author will be given a casting vote. New outcomes will be introduced into the Delphi Survey during the second round.

At second and third rounds of the Delphi Survey, panellists will be provided with median scores for the whole group and a graphical representation of the score and interquartile ranges. Individual stakeholder group sub-group analysis will not be performed here but will be performed and provided to participants in the consensus meeting. All panellists that submit a full or partial response will be invited to contribute to the next round.

### Which outcomes should be included in the core outcome set?

**Figure 2:**
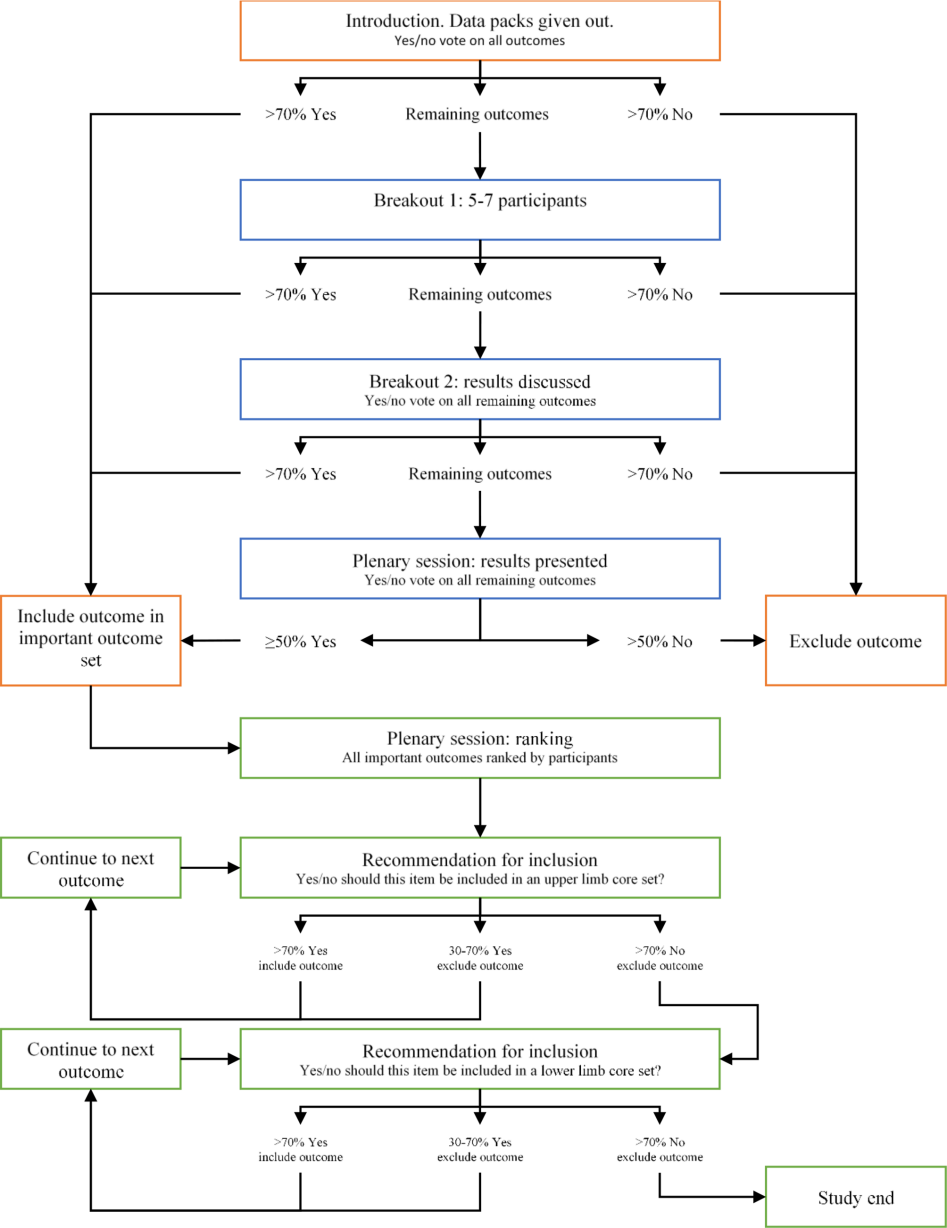
Study flow for consensus meeting. Important outcomes will be identified through voting following each breakout session. Important outcomes will then be ranked and further voting will be completed to identify the outcomes for inclusion in the core outcome set.

The outputs from the three preceding studies will be evaluated at a face to face consensus meeting to develop the core outcome set for use in trials of childhood fractures. The consensus meeting will follow an iterative methodology modelled on the work on developing WHO core sets as reported by Selb *et al*.^37^ The study flow chart is shown in figure 2.

The objective of the meeting will be to identify the most important outcome domains for inclusion I the core outcome set and to develop recommendations for the core outcome set with additional upper and lower limb modules. Expert participants will be approached to represent the following stakeholder groups:

- Parents of children who have had a broken bone.
- Doctors or surgeons who treat children with broken bones.
- Paediatric nurses and therapists.
- Teachers.
- Researchers who have been involved with studies for children with broken bones.
- Systematic reviewers who have been involved with reviews of studies for children with broken bones.

A minimum of 24 participants will be involved in voting and deciding on the outcome set to permit breakout sessions of 7 participants for facilitated discussion of each outcome domain.

Participants will be provided with an information pack summarising the findings from the first three phases of this study. An initial in/out screening vote on each outcome will be performed at the start of the meeting to reduce the number of outcome domains that require discussion in the breakout sessions. Further voting will follow facilitated breakout sessions where remaining outcome domains will be evaluated and discussed. Voting will be performed using an electronic survey interface to enable real time analysis and rapid feedback to participants.

At each voting round, outcome domains will be included or excluded if the consensus threshold of 70% is met. All remaining outcome domains will be discussed in facilitated breakout sessions. Two rounds of discussion are planned followed by majority voting to ensure that all outcome domains are categorised for further discussion or exclusion.

Once an important outcome domain set is derived, participants will score all outcome domains to produce a ranked list of outcomes for upper and lower limb fractures. Voting will occur in order of outcome rank to identify those for the upper limb core set (>70% ‘Yes’). When 30%–70% of participants vote ‘Yes’ the outcome domain will be identified as important, but not core. When >30% vote ‘No’ then the round will close, and the ranked voting will be repeated for lower limb outcome domains.

Outcome domains that are voted for inclusion in both the upper and lower limb core sets will form the generic fracture core set. Outcome domains voted for inclusion in the upper limb but not the lower limb core set will form the upper limb module and outcome domains voted for inclusion in the lower limb but not the upper limb core set will form the lower limb module.

### Dissemination

Study results will be presented at national and international meetings and disseminated via peer-reviewed journals. Participants will be provided with a plain English summary of results and a statement will be placed on the Trauma Outcome Group’s website.

### Patient and public involvement

Patients and parents have been actively involved in shaping this research as collaborators with the research team. Patient and public involvement has been maintained through all stages of development including defining research question, trial design and selection of methodologies.

## Discussion

The CORE-Kids Study will develop a core outcome set for use in all trials relating to the management of fractures in children. The development of this core outcome set has been scoped to deliver a versatile set of outcomes, with additional modules for upper and lower limb fractures.

The core outcome set will make identification and justification of the selection of outcomes as primary and secondary outcomes easier for future trials and standardise reporting of new trials.

The CORE-Kids outcome set will need development in the future through selection of outcome tools to measure the outcome domains identified in the set. This will require an understanding of the measurement properties of outcome tools to guide selection and recommendation of the best tools to undertake measurements.^51^ This represents important further work that will compliment this study.

## Supplementary Material

Reviewer comments

Author's manuscript
