# Supplementary material for: CORE-Kids: a protocol for the development of a core outcome set for childhood fractures
Source: BMJ Open. 2020 Feb 28;10(2):e036224. doi: 10.1136/bmjopen-2019-036224 (PMC7050303; doi:10.1136/bmjopen-2019-036224)
Supplement: Author's manuscript [file bmjopen-2019-036224.draft_revisions.pdf]

BMJ Open is committed to open peer review. As part of this commitment we make the peer review history of every article we publish publicly available.

When an article is published we post the peer reviewers' comments and the authors' responses online. We also post the versions of the paper that were used during peer review. These are the versions that the peer review comments apply to.

The versions of the paper that follow are the versions that were submitted during the peer review process. They are not the versions of record or the final published versions. They should not be cited or distributed as the published version of this manuscript.

BMJ Open is an open access journal and the full, final, typeset and author-corrected version of record of the manuscript is available on our site with no access controls, subscription charges or pay-per-view fees (http://bmjopen.bmj.com).

If you have any questions on BMJ Open's open peer review process please email info.bmjopen@bmj.com

BMJ Open

CORE-Kids: A protocol for the development of a core outcome set for childhood fractures

Journal:	*BMJ Open*
Manuscript ID	bmjopen-2019-036224
Article Type:	Protocol
Date Submitted by the Author:	05-Dec-2019

Complete List of Authors:	Marson, Ben; University of Nottingham, Trauma Outcomes Group<br>Manning, Joseph; The University of Nottingham, School of Health Sciences<br>James, Marilyn; University of Nottingham, School of Medicine<br>Craxford, Simon; University of Nottingham, Trauma Outcomes Group<br>Deshmukh, Sandeep; University of Nottingham, Trauma Outcomes Group<br>Ollivere, Benjamin; University of Nottingham, Trauma Outcomes Group

Keywords:	Paediatric orthopaedics < ORTHOPAEDIC & TRAUMA SURGERY, Trauma management < ORTHOPAEDIC & TRAUMA SURGERY, QUALITATIVE RESEARCH

SCHOLARONE™
Manuscripts

I, the Submitting Author has the right to grant and does grant on behalf of all authors of the Work (as defined in the below author licence), an exclusive licence and/or a non-exclusive licence for contributions from authors who are: i) UK Crown employees; ii) where BMJ has agreed a CC-BY licence shall apply, and/or iii) in accordance with the terms applicable for US Federal Government officers or employees acting as part of their official duties; on a worldwide, perpetual, irrevocable, royalty-free basis to BMJ Publishing Group Ltd ("BMJ") its licensees and where the relevant Journal is co-owned by BMJ to the co-owners of the Journal, to publish the Work in this journal and any other BMJ products and to exploit all rights, as set out in our licence.

The Submitting Author accepts and understands that any supply made under these terms is made by BMJ to the Submitting Author unless you are acting as an employee on behalf of your employer or a postgraduate student of an affiliated institution which is paying any applicable article publishing charge ("APC") for Open Access articles. Where the Submitting Author wishes to make the Work available on an Open Access basis (and intends to pay the relevant APC), the terms of reuse of such Open Access shall be governed by a Creative Commons licence – details of these licences and which Creative Commons licence will apply to this Work are set out in our licence referred to above.

Other than as permitted in any relevant BMJ Author's Self Archiving Policies, I confirm this Work has not been accepted for publication elsewhere, is not being considered for publication elsewhere and does not duplicate material already published. I confirm all authors consent to publication of this Work and authorise the granting of this licence.

For peer review only

CORE-Kids: A protocol for the development of a core outcome set for childhood fractures

Marson BA, ben.marson@nottingham.ac.uk

Manning JC, joseph.manning@nottingham.ac.uk

James M, Marilyn.james@nottingham.ac.uk

Craxford S, simon.craxford1@nottingham.ac.uk

Deshmukh SR, Sandeep.deshmukh@nottingham.ac.uk

Ollivere BJ Benjamin.ollivere@nottingham.ac.uk

& The CORE-Kids Group

Corresponding author:
Ben Marson
Trauma Outcomes Group
Department of orthopaedics
School of Medicine
University of Nottingham
C-Floor, QMC
Derby Road
Nottingham
NG7 2UH
07779922375

For peer review only

Abstract

Introduction

Limb fractures in children are common yet there are few trials that compare treatments for these injuries. There is significant heterogeneity in the outcomes reported in the paediatric orthopaedic literature, which limits the ability to compare study results and draw firm conclusions. The aim of the CORE-Kids study is to develop a core outcome set for use in research studies of childhood limb fractures.

A core outcome set will provide a minimum set of outcomes to be measured in all trials to minimise the heterogeneity of outcomes reported and minimise reporting bias. A core outcome set ensures outcomes are reported that are relevant to families as well as clinicians. The core outcome set will include additional upper and lower limb modules.

Methods

The development of the core outcome set will require four phases to evaluate:

1. What are the outcomes that are relevant to professionals?
2. What are the outcomes that are relevant to families?
3. What are the most important of these outcomes?
4. Which outcomes should be included in the core outcome set?

This will be completed through a systematic review of trials to identify the outcomes domains that are relevant to trialists. A series of semi-structured interviews will be completed with families to identify the outcome domains relevant to families. These outcome domains will be used in a three round Delphi study to analyse the importance of these outcome domains to a range of stakeholders including parents, clinicians and researchers. Following this, the core outcome set will be decided at a consensus meeting.

Ethics and dissemination

Ethical approval has been awarded HRA/REC IRAS number 262503. Date of approval 06/08/2019. Dissemination will be through scientific literature and international societies.

Strengths and limitations of this study

- This is a core outcome set with a broad scope that will be widely applicable to children's fractures and injuries
- The core outcome set draws from experience from a wide range of stakeholders to ensure the outcomes selected are relevant
- The scope of this study id to deliver an outcome set for children aged 5-15 for randomised trials. It may be applicable to all research studies and to infants and adolescents, but further work will be required to confirm this.

Trial registration

Core Outcome Measures in Effectiveness Trials Initiative (COMET), registration number: 1274 Date of registration 13/12/2018

For peer review only

PROSPERO systematic review registration number: CRD42018106605. Date of registration 21/09/2018

Key words

Fracture, bone; Orthopedic; paediatrics; outcome measure; outcome studies;

Word count

3484 words

For peer review only

Introduction

The World Health Organisation and UNICEF have identified childhood injury as a leading cause of mortality and morbidity in low and middle income countries[1]. The precise global burden of childhood fractures is not known, but 1 in 3 children in Europe will sustain a fracture by their 17th birthday[2].

There are six Cochrane reviews that have evaluated the literature supporting the management of childhood fractures[3–8]. The reviews have identified 45 trials with sufficient quality to inform practice, with most trials relating to the management of wrist fractures. Only two reviews were able to draw firm conclusions due to a paucity of randomised trials in children's fracture care, despite the relative high frequency of these injuries[5,7].

There is a need to deliver high quality randomised trials to evaluate treatments in childhood fractures, and these trials need to be underpinned by a robust rationale for the selection of primary and secondary outcomes[9–11]. There is a growing use of patient reported outcomes in paediatric orthopaedics with little agreement regarding the selection of outcome tools to compare treatments[12,13].

To compare treatments or interventions, an outcome needs to be identified which encompasses a feature of the condition to measure and a technique to measure it. An outcome domain is the broad feature of the condition or a patient's health that is being measured (e.g. pain, range of movement or function), whereas the outcome tool is the technique used to measure it[14].

The heterogeneity of reporting of outcomes can be improved through the development and adoption of a core outcome set[15,16]. This is an agreed minimum standard group of outcomes that are reported in every research study[17]. Through using a core set and adding any additional relevant secondary outcomes, trialists can ensure their results are relevant to surgeons, families and other stakeholders and can be effectively combined for evidence synthesis and guideline generation[14].

The Core Outcome Measures in Effectiveness Trials (COMET) initiative is an international collaboration with the objective of promoting the development and use of core outcome sets. The COMET initiative has published a handbook evaluating the available methodology for developing a core outcome set [18] and reporting standards for the development and reporting of core outcome sets [19,20].

There are only a few core outcome sets that have been developed for use in orthopaedic trials[21]. For trials relating to fractures, the only core outcome set available has been developed for trials of hip fractures in the elderly which would not be appropriate to extrapolate onto fractures in children[22]. A core outcome set is in development for medial epicondyle fractures in children, but this is a relatively uncommon injury and is focussed on a single fracture type[23].

The CORE-Kids study will develop a core outcome set identifying the core domains that should be measured in every trial of childhood fractures. This protocol has been devised in line with the COS-STAP statement [24] and the development studies will be completed and reported as per the COS-STAD and COS-STAR statements [19,20].

Study scope

The scope for this core outcome set has been set by the steering group in conjunction with the NIHR Trauma Trials network.

For peer review only

The scope for this core outcome set is:

- Setting: Research studies;
- Health condition: fractures to the appendicular skeleton (i.e. limbs, pelvis, shoulder girdle but not spine, ribs or head) excluding children with multiple injuries. The core outcome set will be divided into three modules: a central "all fractures" set, an "upper limb" and a "lower limb" set;
- Target population: School aged children (aged 5-16) – it is anticipated that infants (0-4) and older adolescents (17-18) may share common core outcomes but this cannot be assumed and should be confirmed in future work;
- Target interventions: treatment for fractures, both involving surgical and non-surgical (conservative) techniques.

Methods and design

The development of the core outcome set will require four phases to evaluate:

1. What are the outcomes that are relevant to trialists?
2. What are the outcomes that relevant to families?
3. What are the most important of these outcomes?
4. Which outcomes should be included in the core outcome set?

These questions will be answered sequentially through a systematic review of trials, interviews with families, an international online Delphi study and a consensus meeting.

What are the outcomes that are relevant to professionals?

In order to understand the outcomes most relevant to professionals, a systematic review of all trials in childhood fractures will be completed. This systematic review will identify all outcomes that have been reported in trials relating to childhood fractures. The review has been prospectively registered in the PROSPERO database CRD42018106605 [25] and will be reported according to Preferred Reporting Items for Systematic Reviews and Meta-Analyses (PRISMA) statement[26].

Studies that are randomised or quasi-randomised will be included where the majority of the participants are children (age<16) who have sustained fractures to the appendicular skeleton or dislocations to large joints. All interventions that are designed to treat fractures will be considered, with the exclusion of studies that evaluate the efficacy of anaesthetic or analgesia techniques in isolation. Study protocols and trial registrations of randomised or quasi-randomised trials will also be included where outcomes are identified.

English language interventional trials of childhood fractures will be identified by searching electronic database, reviewing bibliographies of Cochrane reviews and hand searching of trial registries and online search engines. The electronic databases that will be searched are OVID MEDLINE, OVID Embase and the Cochrane Central Register of Controlled Trial (CENTRAL). A manual search of clinicaltrials.gov, the ISRCTN registry and WHO ICTRP registry will be completed to identify any unpublished or in-progress trials.

Titles will be screened by one researcher and full texts analysed by two researchers. Outcomes and outcome tools will be extracted and classified according to the World Health Organisation International Classification of functioning (WHO ICF)[27].

For peer review only

No formal assessment of study quality or risk of bias will be completed as the objective of this review is to identify outcomes reported in trials rather than prioritise outcomes or evaluate treatment options. Therefore, the quality of each study will not add any additional value to this review. This is consistent with other systematic review of outcomes reported in different medical and surgical conditions[28–30].

What are the outcomes that are relevant to families?

The outcomes that are relevant to families will be evaluated through a series of semi-structured interviews with parent-child dyads (composed of the child and at least one parent or carer) where the child has recent experience of breaking a bone in the appendicular skeleton. The use of semi-structured interview design has been selected to maximize the depth of the data that will be obtained while ensuring different domains within the WHO international classification of function (WHO ICF) are covered. Interviews will be completed in the fracture clinic as a familiar environment following the child's final clinical review[31,32].

Interviews will be conducted by a PhD research student with supervision from an expert in children's qualitative research. The PhD student will receive training in interview skills and analysis techniques prior to completing these aspects of the study

The sample size has been set to achieve data saturation. A target of 20 interviews will be conducted with parent-child dyads. Parent-child dyads have been selected to provide a familiar adult for the child, to allow the child to express their opinions and obtain the combined experience of parents and children[31,33,34]. Sample size has been determined as a minimum required to achieve saturation after consideration of the scope of identifying outcomes and that the nature of the topic involves discussing issues that are not sensitive or distressing[35].

Heterogeneous, purposeful sampling will be applied to achieve a mix of families with children of different genders, fracture sites (upper and lower limb), fracture severity (inpatient or outpatient treatment) and experience of surgery and experience of cast treatment[36]. Families will be invited to volunteer and participate through posters in the clinic.

A semi-structured interview schedule has been developed based on the recommendations of Selb *et al* [37]. The schedule has been modified following pilot testing with a parent involvement group of families. Interviews will be recorded and transcribed.

As advocated in the literature art-based approaches (draw and tell, graphic elicitation) will be used to facilitate the involvement and verbal responses from child participants[38,39]. The focus of the semi-structured interviews will be on exploring the following areas: (i) experience of injury; (ii) frustrations of recovering and (iii) features of recovery and positive and negative outcomes.

A content analysis will be performed to identify the outcome domains that are identified by parents and children that are relevant to them during the experience of injury and recovery. The outcome domains will be classified according to the WHO ICF based on the linking rules by Cieza *et al.* to maintain consistency with the outcomes that are relevant to trialists identified during the systematic review[40,41]. Coding for the content analysis will be performed by two researchers and a saturation table completed using the identified WHO ICF domains to ensure saturation is achieved prior to completion of the study.

The findings from these interviews will be combined with the results of the systematic review to draw a long list of outcome domains for evaluation using consensus methodology.

For peer review only - http://bmjopen.bmj.com/site/about/guidelines.xhtml

What are the most important of these outcomes?

The long list of candidate outcomes will be reduced through an international three round Delphi survey involving relevant stakeholders. As a method of developing consensus, the Delphi technique involves obtaining successive scores for each outcome from each expert panellist following feedback over multiple survey rounds[42,43].

The Delphi survey has been designed so that each outcome domain will be evaluated twice: once for importance for upper limb trials and once for lower limb trials. All outcomes will be reviewed by our patent participation group to ensure comprehensibility by lay panellists as well as professionals.

A three round design has been selected to maximise the chance of delivering consensus, while minimising the attrition bias that the survey is exposed to if too many rounds are undertaken. In the general medical literature and in the developments of core outcome sets, the median and upper quartile number of rounds completed is three rounds [44,45], with only 6% of Delphi studies used to agree importance of outcomes incorporating more than 3 rounds[46].

As no feedback mechanism has been proven to be most effective in generating consensus[47], we will provide multiple-combined feedback from all groups to minimise the number of results that need to be interpreted by panellists.

The Delphi study will be completed as an online survey using the onlinesurveys.ac.uk interface (JISC, Bristol UK). Panellists will be assigned a unique code to access the online survey system to permit monitoring of survey completion and attrition. All panellists will be sent up to four e-mail reminders of survey deadlines to maximise completion.

Expert panellists will be approached to represent the following stakeholder groups:

- Parents of children who have had a broken bone
- Doctors or surgeons who treat children with broken bones
- Paediatric nurses and therapists
- Teachers
- Researchers who have been involved with studies for children with broken bones
- Systematic reviewers who have been involved with reviews of studies for children with broken bones

Panellists will be assigned a unique code to access the online survey system to permit recording of survey completion and attrition. All panellists will be sent e-mail reminders of survey deadlines to maximise completion.

For each outcome domain, panellists will assign scores from 1 through to 9 (very important) using a Likert scale. Each outcome domain will be scored twice in the first round, once for upper limb and once for lower limb fractures. In subsequent rounds, outcome domains may be scored once or twice, depending if consensus has been reached.

In order to minimise the attrition bias associated with Delphi surveys with higher numbers of items[46], outcome domains will be removed from the second and third rounds if consensus is reached. Outcome domains. Outcome domains will be removed from further Delphi rounds if consensus in (> 70% panellists ranking an outcome with a score of 7-9 and fewer than 15% scoring the outcome 1-3) or consensus out (> 70% panellists ranking an outcome with a score of 1-3 and fewer than 15% scoring the outcome 7-9) is achieved (Figure 1). This consensus threshold has been selected as it is consistent with previous methodology for the development of core outcome

For peer review only

sets[48–50] and ensures outcome domains are only retained or exclude if there is a significant majority voting in favour with a small minority expressing the opposite opinion[17].

No sample size calculations may be performed to guide the panel size for this Delphi. As a target, 75-100 panellists will be recruited to permit for attrition between rounds and to ensure each stakeholder group has representation[18]. The stakeholder groups will be pooled into a single panel for completion of the Delphi survey and in the preparation of feedback between survey rounds. This is to minimise the research burden on panellists and to maximise the likelihood of reaching consensus[18]. A key potential concern with this methodology is that it is possible for the opinion of minority key stakeholder groups to be overwhelmed if the stakeholder balance is not controlled. To prevent this situation, three key stakeholder groups (1. doctors and researchers 2. Nurses and therapists 3. Parents) will each contribute to a target minimum of 20% of the total participants in the panel.

During the first round of Delphi, panellists will be invited to contribute any additional relevant outcomes that have not been captured in the systematic review or qualitative interviews for inclusion in the second-round survey. Outcome domains that are consensus in or remain following the three rounds of Delphi will be included in the consensus meeting for consideration for the core outcome set.

Additional outcomes that are suggested in the first-round survey will be coded by two researchers to ensure that it represents a new outcome. Where there is disagreement between the two researchers, the senior author will be given a casting vote. New outcomes will be introduced into the Delphi survey during the second round.

At second and third round of the Delphi survey, panellists will be provided with median scores for the whole group and a graphical representation of the score and interquartile ranges. Individual stakeholder group sub-group analysis will not be performed here but will be performed and provided to participants in the consensus meeting. All panellists that submit a full or partial response will be invited to contribute to the next round.

For peer review only

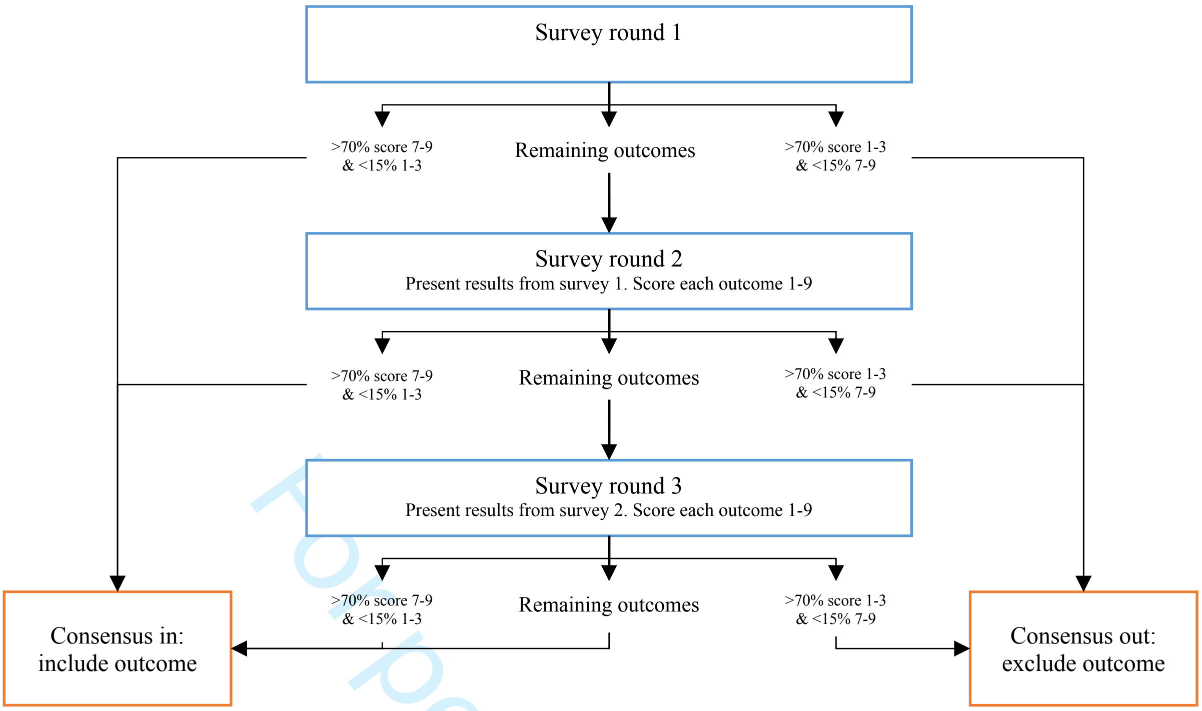

Figure 1 Study flow chart for the three round Delphi study. Outcome domains will be identified as 'consensus in' if 70% of panellists rate the outcome domain with a score of 7-9 and fewer than 15% of panellists score the domain 1-3. Outcome domains will be identified as 'consensus out' if 70% of panellists score the outcome domain 1-3 and fewer than 15% of panellists score the domain 7-9.

Which outcomes should be included in the core outcome set?

The outputs from the three preceding studies will be evaluated at a face to face consensus meeting to develop the core outcome set for use in trials of childhood fractures. The consensus meeting will follow an iterative methodology modelled on the work on developing WHO core sets as reported by Selb *et al*. [37] The study flow chart is shown in Figure 2.

The objective of the meeting will be to identify the most important outcome domains for inclusion I the core outcome set and to develop recommendations for the core outcome set with additional upper and lower limb modules. Expert participants will be approached to represent the following stakeholder groups:

- Parents of children who have had a broken bone
- Doctors or surgeons who treat children with broken bones
- Paediatric nurses and therapists
- Teachers
- Researchers who have been involved with studies for children with broken bones
- Systematic reviewers who have been involved with reviews of studies for children with broken bones

A minimum of 24 participants will be involved in voting and deciding on the outcome set to permit breakout sessions of 7 participants for facilitated discussion of each outcome domain.

Participants will be provided with an information pack summarising the findings from the first three phases of this study. An initial in/out screening vote on each outcome will be performed at the start of the meeting to reduce the number of outcome domains that require discussion in the breakout

For peer review only

sessions. Further voting will follow facilitated breakout sessions where remaining outcome domains will be evaluated and discussed. Voting will be performed using an electronic survey interface to enable real time analysis and rapid feedback to participants.

At each voting round, outcome domains will be included or excluded if the consensus threshold of 70% is met. All remaining outcome domains will be discussed in facilitated breakout sessions. Two rounds of discussion are planned followed by majority voting to ensure all outcome domains are categorised for further discussion or exclusion.

Once an important outcome domain set is derived participants will score all outcome domains to produce a ranked list of outcomes for upper and lower limb fractures. Voting will occur in order of outcome rank to identify those for the upper limb core set (>70% 'Yes'). When 30-70% of participants vote 'Yes' the outcome domain will be identified as important, but not core. When >30% vote 'No' then the round will close, and the ranked voting will be repeated for lower limb outcome domains.

Outcome domains that are voted for inclusion in both the upper and lower limb core sets will form the generic fracture core set. Outcome domains voted for inclusion in the upper limb but not the lower limb core set will form the upper limb module and outcome domains voted for inclusion in the lower limb but not the upper limb core set will form the lower limb module.

For peer review only

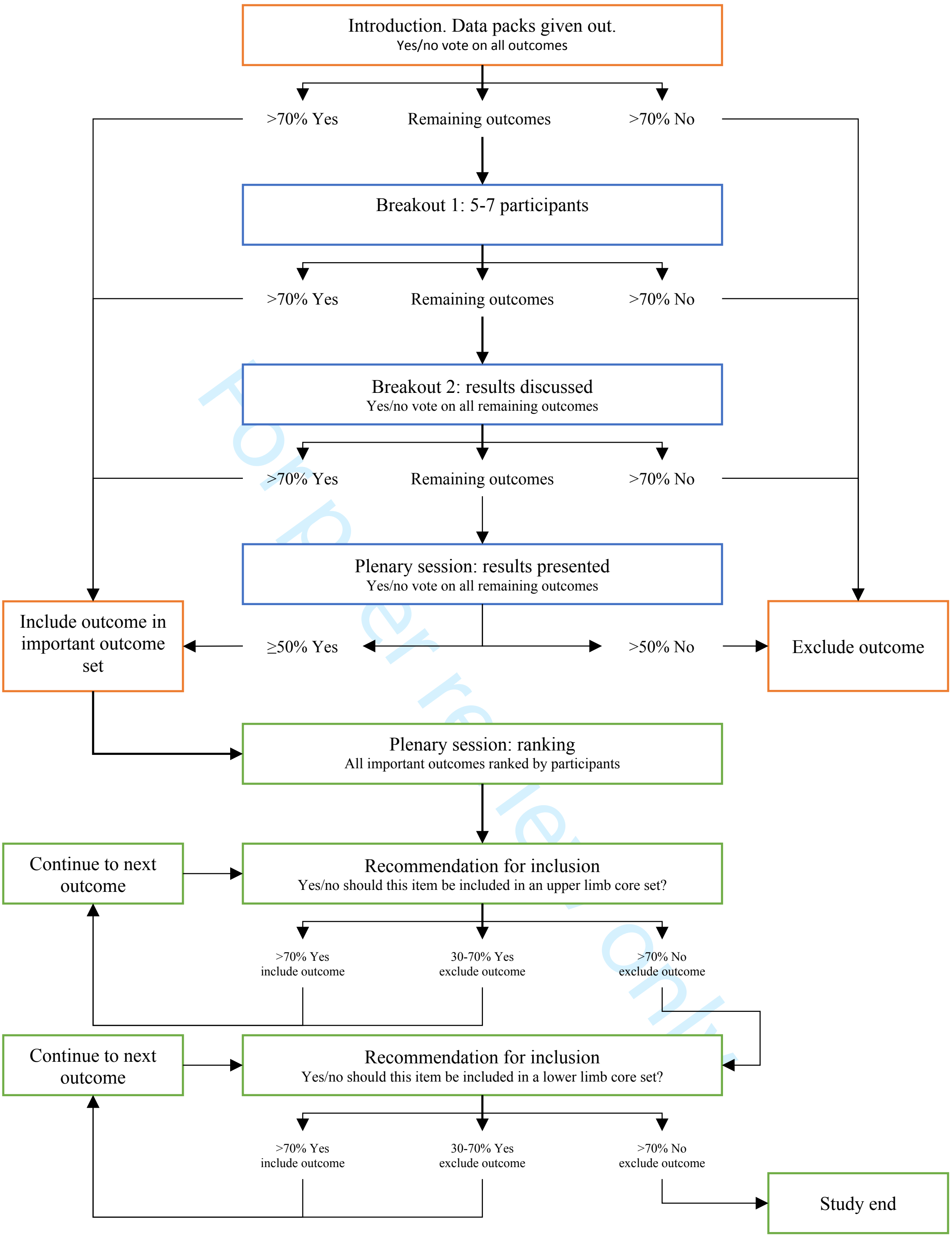

Figure 2 Study flow for consensus meeting. Important outcomes will be identified through voting following each breakout session. Important outcomes will then be ranked and further voting will be completed to identify the outcomes for inclusion in the core outcome set.

Ethics and dissemination

Ethical approval has been awarded on the 06/08/2019 by the North London – Hampstead REC. (HRA/REC IRAS number 262503).

Study results will be presented at national and international meetings and disseminated via peer reviewed journals. Participants will be provided with a plain English summary of results and a statement will be placed on the Trauma Outcome Group's website.

Patient and public involvement

Patients and parents have been actively involved in shaping this research as collaborators with the research team. PPI involvement has been maintained through all stages of development including defining research question, trial design and selection of methodologies.

Discussion

The CORE-Kids study will develop a core outcome set for use in all trials relating to the management of fractures in children. The development of this core outcome set has been scoped to deliver a versatile set of outcomes, with additional modules for upper and lower limb fractures.

The core outcome set will make identification and justification of the selection of outcomes as primary and secondary outcomes easier for future trials and standardise reporting of new trials.

The CORE-Kids outcome set will need development in the future through selection of outcome tools to measure the outcome domains identified in the set. This will require an understanding of the measurement properties of outcome tools to guide selection and recommendation of the best tools to undertake measurements[51]. This represents important further work that will compliment this study.

Conflicts of interest

None of the authors have any conflicts of interest

Contribution of authors

BAM, JM, MJ, BJO conceptualised and designed the overall research project

BAM, SC, SD will complete the systematic review search and extraction from eligible papers

BAM & JM will complete the interviews with families

BAM, SC, SD, MJ and BJO have designed the Delphi strategy

BAM, SC, SD, JM, MJ & BJO will facilitate the consensus meeting

The CORE-kids group will be the key recruiters and disseminators for the study through international networks

BAM, SC, SD, JM, MJ & BJO edited the manuscript and read and approved the final version.

For peer review only

Funding

Ben Marson is funded by a National Institute for Health Research (NIHR), Doctoral Fellowship NIHR300240 for this research project. This report presents independent research funded by the National Institute for Health Research (NIHR). The views expressed are those of the author(s) and not necessarily those of the NHS, the NIHR or the Department of Health and Social Care.

For peer review only

References

1 Peden M, Oyegbite K, Ozanne-Smith J, *et al.* World report on child injury prevention. Geneva: 2008.

2 European Association for Injury Prevention and Safety Promotion. Injuries in the European Union. Amsterdam: 2014.

3 Abraham A, Kumar S, Chaudhry S, *et al.* Surgical interventions for diaphyseal fractures of the radius and ulna in children. In: Abraham A, ed. *Cochrane Database of Systematic Reviews*. Chichester, UK: : John Wiley & Sons, Ltd 2011. doi:10.1002/14651858.CD007907.pub2

4 Madhuri V, Dutt V, Gahukamble AD, *et al.* Conservative interventions for treating diaphyseal fractures of the forearm bones in children. In: Madhuri V, ed. *Cochrane Database of Systematic Reviews*. Chichester, UK: : John Wiley & Sons, Ltd 2013. doi:10.1002/14651858.CD008775.pub2

5 Handoll HH, Elliott J, Iheozor-Ejiofor Z, *et al.* Interventions for treating wrist fractures in children. In: Handoll HH, ed. *Cochrane Database of Systematic Reviews*. Chichester, UK: : John Wiley & Sons, Ltd 2016. doi:10.1002/14651858.CD012470

6 Capstick R, Giele H. Interventions for treating fingertip entrapment injuries in children. In: Capstick R, ed. *Cochrane Database of Systematic Reviews*. Chichester, UK: : John Wiley & Sons, Ltd 2014. doi:10.1002/14651858.CD009808.pub2

7 Yeung DE, Jia X, Miller CA, *et al.* Interventions for treating ankle fractures in children. In: Yeung DE, ed. *Cochrane Database of Systematic Reviews*. Chichester, UK: : John Wiley & Sons, Ltd 2016. doi:10.1002/14651858.CD010836.pub2

8 Madhuri V, Dutt V, Gahukamble AD, *et al.* Interventions for treating femoral shaft fractures in children and adolescents. In: Madhuri V, ed. *Cochrane Database of Systematic Reviews*. Chichester, UK: : John Wiley & Sons, Ltd 2014. doi:10.1002/14651858.CD009076.pub2

9 Ioannidis JPA, Greenland S, Hlatky MA, *et al.* Increasing value and reducing waste in research design, conduct, and analysis. Lancet. 2014;**383**:166–75. doi:10.1016/S0140-6736(13)62227-8

10 Kirkham JJ, Dwan KM, Altman DG, *et al.* The impact of outcome reporting bias in randomised controlled trials on a cohort of systematic reviews. *BMJ* 2010;**340**:637–40. doi:10.1136/bmj.c365

11 Clarke M. Standardising outcomes for clinical trials and systematic reviews. Trials. 2007;**8**. doi:10.1186/1745-6215-8-39

12 Truong WH, Price MJ, Agarwal KN, *et al.* Utilization of a Wide Array of Nonvalidated Outcome Scales in Pediatric Orthopaedic Publications. *J Pediatr Orthop* 2019;**39**:e153–8. doi:10.1097/BPO.0000000000001263

13 Phillips L, Carsen S, Vasireddi A, *et al.* Use of Patient-reported Outcome Measures in Pediatric Orthopaedic Literature. *J Pediatr Orthop* 2018;**38**:393–7. doi:10.1097/BPO.0000000000000847

14 Black N. Patient reported outcome measures could help transform healthcare. *BMJ* 2013;**346**. doi:10.1136/bmj.f167

15 Kirkham JJ, Boers M, Tugwell P, *et al.* Outcome measures in rheumatoid arthritis randomised trials over the last 50 years. *Trials* 2013;**14**:324. doi:10.1186/1745-6215-14-324

For peer review only

16 Kirkham JJ, Clarke M, Williamson PR. A methodological approach for assessing the uptake of core outcome sets using ClinicalTrials.gov: findings from a review of randomised controlled trials of rheumatoid arthritis. *BMJ* 2017;:j2262. doi:10.1136/bmj.j2262

17 Williamson PR, Altman DG, Blazeby JM, *et al.* Developing core outcome sets for clinical trials: Issues to consider. *Trials* 2012;**13**. doi:10.1186/1745-6215-13-132

18 Williamson PR, Altman DG, Bagley H, *et al.* The COMET Handbook: version 1.0. *Trials* 2017;**18**:280. doi:10.1186/s13063-017-1978-4

19 Kirkham JJ, Davis K, Altman DG, *et al.* Core Outcome Set-STAndards for Development: The COS-STAD recommendations. *PLoS Med* 2017;**14**:e1002447. doi:10.1371/journal.pmed.1002447

20 Kirkham JJ, Gorst S, Altman DG, *et al.* Core Outcome Set-STAndards for Reporting: The COS-STAR Statement. *PLoS Med* 2016;**13**:e1002148. doi:10.1371/journal.pmed.1002148

21 Ollivere BJ, Marson BA, Haddad FS. Getting the right answer: core outcome sets in orthopaedics. *Bone Joint J* 2019;**101-B**:233–4. doi:10.1302/0301-620X.101B3.BJJ-2019-0065

22 Haywood KL, Griffin XL, Achten J, *et al.* Developing a core outcome set for hip fracture trials. *Bone Joint J* 2014;**96-B**:1016–23. doi:10.1302/0301-620X.96B8.33766

23 Dorman SL, Shelton JA, Stevenson RA, *et al.* Management of medial humeral epicondyle fractures in children: a structured review protocol for a systematic review of the literature and identification of a core outcome set using a Delphi survey. Trials. 2018;**19**:119. doi:10.1186/s13063-018-2472-3

24 Kirkham JJ, Gorst S, Altman DG, *et al.* Core Outcome Set-STAndardised Protocol Items: the COS-STAP Statement. *Trials* 2019;**20**:116. doi:10.1186/s13063-019-3230-x

25 Marson BA, Deshmukh SR, Grindlay D, *et al.* A systematic review of outcomes measured in trials of treatments for fractures in children. PROSPERO 2018: CRD42018106605. PROSPERO. 2018.http://www.crd.york.ac.uk/PROSPERO/display_record.php?ID=CRD42018106605 (accessed 3 Oct 2018).

26 Moher D, Liberati A, Tetzlaff J, *et al.* Preferred Reporting Items for Systematic Reviews and Meta-Analyses: The PRISMA Statement. *PLoS Med* 2009;**6**:e1000097. doi:10.1371/journal.pmed.1000097

27 WHO. International Classification of Functioning, Disability and Health (ICF). WHO. 2001.http://www.who.int/classifications/icf/en/ (accessed 5 Sep 2018).

28 Young AE, Davies A, Bland S, *et al.* Systematic review of clinical outcome reporting in randomised controlled trials of burn care. BMJ Open. 2019;**9**. doi:10.1136/bmjopen-2018-025135

29 A. M, N. B, J. M, *et al.* Toward a Core Outcome Set for Head, Neck, and Respiratory Disease in Mucopolysaccharidosis Type II: Systematic Literature Review and Assessment of Heterogeneity in Outcome Reporting. *J Inborn Errors Metab Screen* 2019;**7**. doi:10.1177/2326409818816721

30 Hancox JM, Toman E, Brace-McDonnell SJ, *et al.* Patient-centred outcomes for prehospital trauma trials: A systematic review and patient involvement exercise. *Trauma (United Kingdom)* Published Online First: 1 October 2019. doi:10.1177/1460408618817912

31 O'Reilly M, Dogra N. Planning your Interview: Key Decisions and Practical Issues. In: O'Reilly M, Dogra N, eds. *Interviewing Children and Young People for Research*. 1 Oliver's Yard, 55 City

For peer review only

Road London EC1Y 1SP: : SAGE Publications Ltd 2017. 71–94. doi:10.4135/9781526419439.n5

32 Bernheimer LP. The use of qualitative methodology in child health research. *Child Health Care* 1986;**14**:224–32. doi:10.1207/s15326888chc1404_6

33 Zajac R, Brown DA. Conducting Successful Memory Interviews with Children. *Child Adolesc Soc Work J* 2018;**35**:297–308. doi:10.1007/s10560-017-0527-z

34 Larsson AS, Lamb ME. Making the most of information-gathering interviews with children. *Infant Child Dev* 2009;**18**:1–16. doi:10.1002/icd.573

35 Morse JM. Determining Sample Size. *Qual Health Res* 2000;**10**:3–5. doi:10.1177/104973200129118183

36 Holloway I, Galvin K. Sampling Stratergies. In: Holloway I, Galvin K, eds. *Qualitative research in nursing and healthcare*. Hoboken: : John Wiley & Sons, Incorporated 2016. 141–56.

37 Selb M, Escorpizo R, Kostanjsek N, *et al.* A guide on how to develop an International Classification of Functioning, Disability and Health Core Set. *Eur J Phys Rehabil Med* 2015;**51**:105–17.

38 Manning JC, Hemingway P, Redsell SA. Stories of survival: Children's narratives of psychosocial well-being following paediatric critical illness or injury. *J Child Heal Care* 2017;**21**:236–52. doi:10.1177/1367493517717078

39 Coad J. Using art-based techniques in engaging children and young people in health care consultations and/or research. *J Res Nurs* 2007;**12**:487–97. doi:10.1177/1744987107081250

40 Cieza A, Fayed N, Bickenbach J, *et al.* Refinements of the ICF Linking Rules to strengthen their potential for establishing comparability of health information. *Disabil Rehabil* 2016;**8288**:1–10. doi:10.3109/09638288.2016.1145258

41 Cieza A, Geyh S, Chatterji S, *et al.* ICF linking rules: an update based on lessons learned. *J Rehabil Med* 2005;**37**:212–8. doi:10.1080/16501970510040263

42 Jones J, Hunter D. Consensus methods for medical and health services research. *BMJ* 1995;**311**:376–80. doi:10.1136/bmj.311.7001.376

43 Helmer-Hirschberg O. Analysis of the future: the Delphi Method. Santa Monica, CA: 1967.

44 Boulkedid R, Abdoul H, Loustau M, *et al.* Using and reporting the Delphi method for selecting healthcare quality indicators: a systematic review. *PLoS One* 2011;**6**:e20476. doi:10.1371/journal.pone.0020476

45 Sinha IP, Smyth RL, Williamson PR. Using the Delphi Technique to Determine Which Outcomes to Measure in Clinical Trials: Recommendations for the Future Based on a Systematic Review of Existing Studies. *PLoS Med* 2011;**8**:e1000393. doi:10.1371/journal.pmed.1000393

46 Gargon E, Crew R, Burnside G, *et al.* Higher number of items associated with significantly lower response rates in COS Delphi surveys. *J Clin Epidemiol* 2019;**108**:110–20. doi:10.1016/J.JCLINEPI.2018.12.010

47 MacLennan S, Kirkham J, Lam TBL, *et al.* A randomized trial comparing three Delphi feedback strategies found no evidence of a difference in a setting with high initial agreement. *J Clin Epidemiol* 2018;**93**:1–8. doi:10.1016/j.jclinepi.2017.09.024

48 Blazeby JM, Macefield R, Blencowe NS, *et al.* Core information set for oesophageal cancer

For peer review only

surgery. *Br J Surg* 2015;**102**:936–43. doi:10.1002/bjs.9840

49 Potter S, Holcombe C, Ward JA, *et al.* Development of a core outcome set for research and audit studies in reconstructive breast surgery. *Br J Surg* 2015;**102**:1360–71. doi:10.1002/bjs.9883

50 Harman NL, Bruce IA, Kirkham JJ, *et al.* The importance of integration of stakeholder views in core outcome set development: Otitis Media with Effusion in children with cleft palate. *PLoS One* 2015;**10**:1–22. doi:10.1371/journal.pone.0129514

51 Prinsen CAC, Vohra S, Rose MR, *et al.* How to select outcome measurement instruments for outcomes included in a " Core Outcome Set " – a practical guideline. *Trials* 2016;**17**. doi:10.1186/s13063-016-1555-2

For peer review only

BMJ Open

CORE-Kids: A protocol for the development of a core outcome set for childhood fractures

Journal:	*BMJ Open*
Manuscript ID	bmjopen-2019-036224.R1
Article Type:	Protocol
Date Submitted by the Author:	19-Dec-2019
Complete List of Authors:	Marson, Ben; University of Nottingham, Trauma Outcomes Group<br>Manning, Joseph; The University of Nottingham, School of Health Sciences<br>James, Marilyn; University of Nottingham, School of Medicine<br>Craxford, Simon; University of Nottingham, Trauma Outcomes Group<br>Deshmukh, Sandeep; University of Nottingham, Trauma Outcomes Group<br>Ollivere, Benjamin; University of Nottingham, Trauma Outcomes Group
**Primary Subject Heading**:	Surgery
Secondary Subject Heading:	Paediatrics
Keywords:	Paediatric orthopaedics < ORTHOPAEDIC & TRAUMA SURGERY, Trauma management < ORTHOPAEDIC & TRAUMA SURGERY, QUALITATIVE RESEARCH

SCHOLARONE™
Manuscripts

I, the Submitting Author has the right to grant and does grant on behalf of all authors of the Work (as defined in the below author licence), an exclusive licence and/or a non-exclusive licence for contributions from authors who are: i) UK Crown employees; ii) where BMJ has agreed a CC-BY licence shall apply, and/or iii) in accordance with the terms applicable for US Federal Government officers or employees acting as part of their official duties; on a worldwide, perpetual, irrevocable, royalty-free basis to BMJ Publishing Group Ltd ("BMJ") its licensees and where the relevant Journal is co-owned by BMJ to the co-owners of the Journal, to publish the Work in this journal and any other BMJ products and to exploit all rights, as set out in our licence.

The Submitting Author accepts and understands that any supply made under these terms is made by BMJ to the Submitting Author unless you are acting as an employee on behalf of your employer or a postgraduate student of an affiliated institution which is paying any applicable article publishing charge ("APC") for Open Access articles. Where the Submitting Author wishes to make the Work available on an Open Access basis (and intends to pay the relevant APC), the terms of reuse of such Open Access shall be governed by a Creative Commons licence – details of these licences and which Creative Commons licence will apply to this Work are set out in our licence referred to above.

Other than as permitted in any relevant BMJ Author's Self Archiving Policies, I confirm this Work has not been accepted for publication elsewhere, is not being considered for publication elsewhere and does not duplicate material already published. I confirm all authors consent to publication of this Work and authorise the granting of this licence.

For peer review only

CORE-Kids: A protocol for the development of a core outcome set for childhood fractures

Marson BA, ben.marson@nottingham.ac.uk Trauma Outcomes Group, Department of orthopaedics, School of Medicine, University of Nottingham NG7 2UH

Manning JC, joseph.manning@nottingham.ac.uk Children & Young People's Health Research (CYP-HR) Group, School of Health Sciences, University of Nottingham

James M, Marilyn.james@nottingham.ac.uk Division of Rehabilitation and Ageing, School of Medicine, Queens Medical Centre, University of Nottingham, Nottingham, NG7 2UH

Craxford S, simon.craxford1@nottingham.ac.uk Trauma Outcomes Group, Department of orthopaedics, School of Medicine, University of Nottingham NG7 2UH
Deshmukh SR, Sandeep.deshmukh@nottingham.ac.uk Trauma Outcomes Group, Department of orthopaedics, School of Medicine, University of Nottingham NG7 2UH

Ollivere BJ Benjamin.ollivere@nottingham.ac.uk Trauma Outcomes Group, Department of orthopaedics, School of Medicine, University of Nottingham NG7 2UH

& The CORE-Kids Group

Corresponding author:
Ben Marson
Trauma Outcomes Group
Department of orthopaedics
School of Medicine
University of Nottingham
C-Floor, QMC
Derby Road
Nottingham
NG7 2UH
0115 924 9924.

For peer review only

Abstract

Introduction

Limb fractures in children are common yet there are few trials that compare treatments for these injuries. There is significant heterogeneity in the outcomes reported in the paediatric orthopaedic literature, which limits the ability to compare study results and draw firm conclusions. The aim of the CORE-Kids study is to develop a core outcome set for use in research studies of childhood limb fractures.

A core outcome set will provide a minimum set of outcomes to be measured in all trials to minimise the heterogeneity of outcomes reported and minimise reporting bias. A core outcome set ensures outcomes are reported that are relevant to families as well as clinicians. The core outcome set will include additional upper and lower limb modules.

Methods

The development of the core outcome set will require four phases to evaluate:

1. What are the outcomes that are relevant to professionals?
2. What are the outcomes that are relevant to families?
3. What are the most important of these outcomes?
4. Which outcomes should be included in the core outcome set?

This will be completed through a systematic review of trials to identify the outcomes domains that are relevant to trialists. A series of semi-structured interviews will be completed with families to identify the outcome domains relevant to families. These outcome domains will be used in a three round Delphi study to analyse the importance of these outcome domains to a range of stakeholders including parents, clinicians and researchers. Following this, the core outcome set will be decided at a consensus meeting.

Ethics and dissemination

Ethical approval has been awarded HRA/REC IRAS number 262503. Date of approval 06/08/2019. Dissemination will be through scientific literature and international societies.

Strengths and limitations of this study

- This is a core outcome set with a broad scope that will be widely applicable to children's fractures and injuries
- The core outcome set draws from experience from a wide range of stakeholders to ensure the outcomes selected are relevant
- The scope of this study id to deliver an outcome set for children aged 5-15 for randomised trials. It may be applicable to all research studies and to infants and adolescents, but further work will be required to confirm this.

Trial registration

Core Outcome Measures in Effectiveness Trials Initiative (COMET), registration number: 1274 Date of registration 13/12/2018

For peer review only

PROSPERO systematic review registration number: CRD42018106605. Date of registration 21/09/2018

Key words

Fracture, bone; Orthopedic; paediatrics; outcome measure; outcome studies;

Word count

3484 words

For peer review only

For peer review only - http://bmjopen.bmj.com/site/about/guidelines.xhtml

Introduction

The World Health Organisation and UNICEF have identified childhood injury as a leading cause of mortality and morbidity in low and middle income countries[1]. The precise global burden of childhood fractures is not known, but 1 in 3 children in Europe will sustain a fracture by their 17th birthday[2].

There are six Cochrane reviews that have evaluated the literature supporting the management of childhood fractures[3–8]. The reviews have identified 45 trials with sufficient quality to inform practice, with most trials relating to the management of wrist fractures. Only two reviews were able to draw firm conclusions due to a paucity of randomised trials in children's fracture care, despite the relative high frequency of these injuries[5,7].

There is a need to deliver high quality randomised trials to evaluate treatments in childhood fractures, and these trials need to be underpinned by a robust rationale for the selection of primary and secondary outcomes[9–11]. There is a growing use of patient reported outcomes in paediatric orthopaedics with little agreement regarding the selection of outcome tools to compare treatments[12,13].

To compare treatments or interventions, an outcome needs to be identified which encompasses a feature of the condition to measure and a technique to measure it. An outcome domain is the broad feature of the condition or a patient's health that is being measured (e.g. pain, range of movement or function), whereas the outcome tool is the technique used to measure it[14].

The heterogeneity of reporting of outcomes can be improved through the development and adoption of a core outcome set[15,16]. This is an agreed minimum standard group of outcomes that are reported in every research study[17]. Through using a core set and adding any additional relevant secondary outcomes, trialists can ensure their results are relevant to surgeons, families and other stakeholders and can be effectively combined for evidence synthesis and guideline generation[14].

The Core Outcome Measures in Effectiveness Trials (COMET) initiative is an international collaboration with the objective of promoting the development and use of core outcome sets. The COMET initiative has published a handbook evaluating the available methodology for developing a core outcome set [18] and reporting standards for the development and reporting of core outcome sets [19,20].

There are only a few core outcome sets that have been developed for use in orthopaedic trials[21]. For trials relating to fractures, the only core outcome set available has been developed for trials of hip fractures in the elderly which would not be appropriate to extrapolate onto fractures in children[22]. A core outcome set is in development for medial epicondyle fractures in children, but this is a relatively uncommon injury and is focussed on a single fracture type[23].

The CORE-Kids study will develop a core outcome set identifying the core domains that should be measured in every trial of childhood fractures. This protocol has been devised in line with the COS-STAP statement [24] and the development studies will be completed and reported as per the COS-STAD and COS-STAR statements [19,20].

Study scope

The scope for this core outcome set has been set by the steering group in conjunction with the NIHR Trauma Trials network.

For peer review only - http://bmjopen.bmj.com/site/about/guidelines.xhtml

The scope for this core outcome set is:

- Setting: Research studies;
- Health condition: fractures to the appendicular skeleton (i.e. limbs, pelvis, shoulder girdle but not spine, ribs or head) excluding children with multiple injuries. The core outcome set will be divided into three modules: a central “all fractures” set, an “upper limb” and a “lower limb” set;
- Target population: School aged children (aged 5-16) – it is anticipated that infants (0-4) and older adolescents (17-18) may share common core outcomes but this cannot be assumed and should be confirmed in future work;
- Target interventions: treatment for fractures, both involving surgical and non-surgical (conservative) techniques.

Methods and design

The development of the core outcome set will require four phases to evaluate:

1. What are the outcomes that are relevant to trialists?
2. What are the outcomes that relevant to families?
3. What are the most important of these outcomes?
4. Which outcomes should be included in the core outcome set?

These questions will be answered sequentially through a systematic review of trials, interviews with families, an international online Delphi study and a consensus meeting.

What are the outcomes that are relevant to professionals?

In order to understand the outcomes most relevant to professionals, a systematic review of all trials in childhood fractures will be completed. This systematic review will identify all outcomes that have been reported in trials relating to childhood fractures. The review has been prospectively registered in the PROSPERO database CRD42018106605 [25] and will be reported according to Preferred Reporting Items for Systematic Reviews and Meta-Analyses (PRISMA) statement[26].

Studies that are randomised or quasi-randomised will be included where the majority of the participants are children (age<16) who have sustained fractures to the appendicular skeleton or dislocations to large joints. All interventions that are designed to treat fractures will be considered, with the exclusion of studies that evaluate the efficacy of anaesthetic or analgesia techniques in isolation. Study protocols and trial registrations of randomised or quasi-randomised trials will also be included where outcomes are identified.

English language interventional trials of childhood fractures will be identified by searching electronic database, reviewing bibliographies of Cochrane reviews and hand searching of trial registries and online search engines. The electronic databases that will be searched are OVID MEDLINE, OVID Embase and the Cochrane Central Register of Controlled Trial (CENTRAL). A manual search of clinicaltrials.gov, the ISRCTN registry and WHO ICTRP registry will be completed to identify any unpublished or in-progress trials.

Titles will be screened by one researcher and full texts analysed by two researchers. Outcomes and outcome tools will be extracted and classified according to the World Health Organisation International Classification of functioning (WHO ICF)[27].

For peer review only - http://bmjopen.bmj.com/site/about/guidelines.xhtml

No formal assessment of study quality or risk of bias will be completed as the objective of this review is to identify outcomes reported in trials rather than prioritise outcomes or evaluate treatment options. Therefore, the quality of each study will not add any additional value to this review. This is consistent with other systematic review of outcomes reported in different medical and surgical conditions[28–30].

What are the outcomes that are relevant to families?

The outcomes that are relevant to families will be evaluated through a series of semi-structured interviews with parent-child dyads (composed of the child and at least one parent or carer) where the child has recent experience of breaking a bone in the appendicular skeleton. The use of semi-structured interview design has been selected to maximize the depth of the data that will be obtained while ensuring different domains within the WHO international classification of function (WHO ICF) are covered. Interviews will be completed in the fracture clinic as a familiar environment following the child's final clinical review[31,32].

Interviews will be conducted by a PhD research student with supervision from an expert in children's qualitative research. The PhD student will receive training in interview skills and analysis techniques prior to completing these aspects of the study

The sample size has been set to achieve data saturation. A target of 20 interviews will be conducted with parent-child dyads. Parent-child dyads have been selected to provide a familiar adult for the child, to allow the child to express their opinions and obtain the combined experience of parents and children[31,33,34]. Sample size has been determined as a minimum required to achieve saturation after consideration of the scope of identifying outcomes and that the nature of the topic involves discussing issues that are not sensitive or distressing[35].

Heterogeneous, purposeful sampling will be applied to achieve a mix of families with children of different genders, fracture sites (upper and lower limb), fracture severity (inpatient or outpatient treatment) and experience of surgery and experience of cast treatment[36]. Families will be invited to volunteer and participate through posters in the clinic.

A semi-structured interview schedule has been developed based on the recommendations of Selb *et al* [37]. The schedule has been modified following pilot testing with a parent involvement group of families. Interviews will be recorded and transcribed.

As advocated in the literature art-based approaches (draw and tell, graphic elicitation) will be used to facilitate the involvement and verbal responses from child participants[38,39]. The focus of the semi-structured interviews will be on exploring the following areas: (i) experience of injury; (ii) frustrations of recovering and (iii) features of recovery and positive and negative outcomes.

A content analysis will be performed to identify the outcome domains that are identified by parents and children that are relevant to them during the experience of injury and recovery. The outcome domains will be classified according to the WHO ICF based on the linking rules by Cieza *et al.* to maintain consistency with the outcomes that are relevant to trialists identified during the systematic review[40,41]. Coding for the content analysis will be performed by two researchers and a saturation table completed using the identified WHO ICF domains to ensure saturation is achieved prior to completion of the study.

The findings from these interviews will be combined with the results of the systematic review to draw a long list of outcome domains for evaluation using consensus methodology.

For peer review only - http://bmjopen.bmj.com/site/about/guidelines.xhtml

What are the most important of these outcomes?

The long list of candidate outcomes will be reduced through an international three round Delphi survey involving relevant stakeholders. As a method of developing consensus, the Delphi technique involves obtaining successive scores for each outcome from each expert panellist following feedback over multiple survey rounds[42,43].

The Delphi survey has been designed so that each outcome domain will be evaluated twice: once for importance for upper limb trials and once for lower limb trials. All outcomes will be reviewed by our patent participation group to ensure comprehensibility by lay panellists as well as professionals.

A three round design has been selected to maximise the chance of delivering consensus, while minimising the attrition bias that the survey is exposed to if too many rounds are undertaken. In the general medical literature and in the developments of core outcome sets, the median and upper quartile number of rounds completed is three rounds [44,45], with only 6% of Delphi studies used to agree importance of outcomes incorporating more than 3 rounds[46].

As no feedback mechanism has been proven to be most effective in generating consensus[47], we will provide multiple-combined feedback from all groups to minimise the number of results that need to be interpreted by panellists.

The Delphi study will be completed as an online survey using the onlinesurveys.ac.uk interface (JISC, Bristol UK). Panellists will be assigned a unique code to access the online survey system to permit monitoring of survey completion and attrition. All panellists will be sent up to four e-mail reminders of survey deadlines to maximise completion.

Expert panellists will be approached to represent the following stakeholder groups:

- Parents of children who have had a broken bone
- Doctors or surgeons who treat children with broken bones
- Paediatric nurses and therapists
- Teachers
- Researchers who have been involved with studies for children with broken bones
- Systematic reviewers who have been involved with reviews of studies for children with broken bones

Panellists will be assigned a unique code to access the online survey system to permit recording of survey completion and attrition. All panellists will be sent e-mail reminders of survey deadlines to maximise completion.

For each outcome domain, panellists will assign scores from 1 through to 9 (very important) using a Likert scale. Each outcome domain will be scored twice in the first round, once for upper limb and once for lower limb fractures. In subsequent rounds, outcome domains may be scored once or twice, depending if consensus has been reached.

In order to minimise the attrition bias associated with Delphi surveys with higher numbers of items[46], outcome domains will be removed from the second and third rounds if consensus is reached. Outcome domains. Outcome domains will be removed from further Delphi rounds if consensus in (> 70% panellists ranking an outcome with a score of 7-9 and fewer than 15% scoring the outcome 1-3) or consensus out (> 70% panellists ranking an outcome with a score of 1-3 and fewer than 15% scoring the outcome 7-9) is achieved (Figure 1). This consensus threshold has been selected as it is consistent with previous methodology for the development of core outcome

For peer review only - http://bmjopen.bmj.com/site/about/guidelines.xhtml

sets[48–50] and ensures outcome domains are only retained or exclude if there is a significant majority voting in favour with a small minority expressing the opposite opinion[17].

No sample size calculations may be performed to guide the panel size for this Delphi. As a target, 75-100 panellists will be recruited to permit for attrition between rounds and to ensure each stakeholder group has representation[18]. The stakeholder groups will be pooled into a single panel for completion of the Delphi survey and in the preparation of feedback between survey rounds. This is to minimise the research burden on panellists and to maximise the likelihood of reaching consensus[18]. A key potential concern with this methodology is that it is possible for the opinion of minority key stakeholder groups to be overwhelmed if the stakeholder balance is not controlled. To prevent this situation, three key stakeholder groups (1. doctors and researchers 2. Nurses and therapists 3. Parents) will each contribute to a target minimum of 20% of the total participants in the panel.

During the first round of Delphi, panellists will be invited to contribute any additional relevant outcomes that have not been captured in the systematic review or qualitative interviews for inclusion in the second-round survey. Outcome domains that are consensus in or remain following the three rounds of Delphi will be included in the consensus meeting for consideration for the core outcome set.

Additional outcomes that are suggested in the first-round survey will be coded by two researchers to ensure that it represents a new outcome. Where there is disagreement between the two researchers, the senior author will be given a casting vote. New outcomes will be introduced into the Delphi survey during the second round.

At second and third round of the Delphi survey, panellists will be provided with median scores for the whole group and a graphical representation of the score and interquartile ranges. Individual stakeholder group sub-group analysis will not be performed here but will be performed and provided to participants in the consensus meeting. All panellists that submit a full or partial response will be invited to contribute to the next round.

Which outcomes should be included in the core outcome set?

The outputs from the three preceding studies will be evaluated at a face to face consensus meeting to develop the core outcome set for use in trials of childhood fractures. The consensus meeting will follow an iterative methodology modelled on the work on developing WHO core sets as reported by Selb *et al*. [37] The study flow chart is shown in **Error! Reference source not found.**.

The objective of the meeting will be to identify the most important outcome domains for inclusion I the core outcome set and to develop recommendations for the core outcome set with additional upper and lower limb modules. Expert participants will be approached to represent the following stakeholder groups:

- Parents of children who have had a broken bone
- Doctors or surgeons who treat children with broken bones
- Paediatric nurses and therapists
- Teachers
- Researchers who have been involved with studies for children with broken bones
- Systematic reviewers who have been involved with reviews of studies for children with broken bones

For peer review only - http://bmjopen.bmj.com/site/about/guidelines.xhtml

A minimum of 24 participants will be involved in voting and deciding on the outcome set to permit breakout sessions of 7 participants for facilitated discussion of each outcome domain.

Participants will be provided with an information pack summarising the findings from the first three phases of this study. An initial in/out screening vote on each outcome will be performed at the start of the meeting to reduce the number of outcome domains that require discussion in the breakout sessions. Further voting will follow facilitated breakout sessions where remaining outcome domains will be evaluated and discussed. Voting will be performed using an electronic survey interface to enable real time analysis and rapid feedback to participants.

At each voting round, outcome domains will be included or excluded if the consensus threshold of 70% is met. All remaining outcome domains will be discussed in facilitated breakout sessions. Two rounds of discussion are planned followed by majority voting to ensure all outcome domains are categorised for further discussion or exclusion.

Once an important outcome domain set is derived participants will score all outcome domains to produce a ranked list of outcomes for upper and lower limb fractures. Voting will occur in order of outcome rank to identify those for the upper limb core set (>70% 'Yes'). When 30-70% of participants vote 'Yes' the outcome domain will be identified as important, but not core. When >30% vote 'No' then the round will close, and the ranked voting will be repeated for lower limb outcome domains.

Outcome domains that are voted for inclusion in both the upper and lower limb core sets will form the generic fracture core set. Outcome domains voted for inclusion in the upper limb but not the lower limb core set will form the upper limb module and outcome domains voted for inclusion in the lower limb but not the upper limb core set will form the lower limb module.

For peer review only

Ethics and dissemination

Ethical approval has been awarded on the 06/08/2019 by the North London – Hampstead REC. (HRA/REC IRAS number 262503).

Study results will be presented at national and international meetings and disseminated via peer reviewed journals. Participants will be provided with a plain English summary of results and a statement will be placed on the Trauma Outcome Group's website.

Patient and public involvement

Patients and parents have been actively involved in shaping this research as collaborators with the research team. PPI involvement has been maintained through all stages of development including defining research question, trial design and selection of methodologies.

Discussion

The CORE-Kids study will develop a core outcome set for use in all trials relating to the management of fractures in children. The development of this core outcome set has been scoped to deliver a versatile set of outcomes, with additional modules for upper and lower limb fractures.

The core outcome set will make identification and justification of the selection of outcomes as primary and secondary outcomes easier for future trials and standardise reporting of new trials.

The CORE-Kids outcome set will need development in the future through selection of outcome tools to measure the outcome domains identified in the set. This will require an understanding of the measurement properties of outcome tools to guide selection and recommendation of the best tools to undertake measurements[51]. This represents important further work that will compliment this study.

Conflicts of interest

None of the authors have any conflicts of interest

Contribution of authors

BAM, JM, MJ, BJO conceptualised and designed the overall research project

BAM, SC, SD will complete the systematic review search and extraction from eligible papers

BAM & JM will complete the interviews with families

BAM, SC, SD, MJ and BJO have designed the Delphi strategy

BAM, SC, SD, JM, MJ & BJO will facilitate the consensus meeting

The CORE-kids group will be the key recruiters and disseminators for the study through international networks

BAM, SC, SD, JM, MJ & BJO edited the manuscript and read and approved the final version.

For peer review only - http://bmjopen.bmj.com/site/about/guidelines.xhtml

Funding

Ben Marson is funded by a National Institute for Health Research (NIHR), Doctoral Fellowship NIHR300240 for this research project. This report presents independent research funded by the National Institute for Health Research (NIHR). The views expressed are those of the author(s) and not necessarily those of the NHS, the NIHR or the Department of Health and Social Care.

For peer review only

For peer review only - http://bmjopen.bmj.com/site/about/guidelines.xhtml

References

1 Peden M, Oyegbite K, Ozanne-Smith J, *et al.* World report on child injury prevention. Geneva: 2008.

2 European Association for Injury Prevention and Safety Promotion. Injuries in the European Union. Amsterdam: 2014.

3 Abraham A, Kumar S, Chaudhry S, *et al.* Surgical interventions for diaphyseal fractures of the radius and ulna in children. In: Abraham A, ed. *Cochrane Database of Systematic Reviews*. Chichester, UK: : John Wiley & Sons, Ltd 2011. doi:10.1002/14651858.CD007907.pub2

4 Madhuri V, Dutt V, Gahukamble AD, *et al.* Conservative interventions for treating diaphyseal fractures of the forearm bones in children. In: Madhuri V, ed. *Cochrane Database of Systematic Reviews*. Chichester, UK: : John Wiley & Sons, Ltd 2013. doi:10.1002/14651858.CD008775.pub2

5 Handoll HH, Elliott J, Iheozor-Ejiofor Z, *et al.* Interventions for treating wrist fractures in children. In: Handoll HH, ed. *Cochrane Database of Systematic Reviews*. Chichester, UK: : John Wiley & Sons, Ltd 2016. doi:10.1002/14651858.CD012470

6 Capstick R, Giele H. Interventions for treating fingertip entrapment injuries in children. In: Capstick R, ed. *Cochrane Database of Systematic Reviews*. Chichester, UK: : John Wiley & Sons, Ltd 2014. doi:10.1002/14651858.CD009808.pub2

7 Yeung DE, Jia X, Miller CA, *et al.* Interventions for treating ankle fractures in children. In: Yeung DE, ed. *Cochrane Database of Systematic Reviews*. Chichester, UK: : John Wiley & Sons, Ltd 2016. doi:10.1002/14651858.CD010836.pub2

8 Madhuri V, Dutt V, Gahukamble AD, *et al.* Interventions for treating femoral shaft fractures in children and adolescents. In: Madhuri V, ed. *Cochrane Database of Systematic Reviews*. Chichester, UK: : John Wiley & Sons, Ltd 2014. doi:10.1002/14651858.CD009076.pub2

9 Ioannidis JPA, Greenland S, Hlatky MA, *et al.* Increasing value and reducing waste in research design, conduct, and analysis. Lancet. 2014;**383**:166–75. doi:10.1016/S0140-6736(13)62227-8

10 Kirkham JJ, Dwan KM, Altman DG, *et al.* The impact of outcome reporting bias in randomised controlled trials on a cohort of systematic reviews. *BMJ* 2010;**340**:637–40. doi:10.1136/bmj.c365

11 Clarke M. Standardising outcomes for clinical trials and systematic reviews. Trials. 2007;**8**. doi:10.1186/1745-6215-8-39

12 Truong WH, Price MJ, Agarwal KN, *et al.* Utilization of a Wide Array of Nonvalidated Outcome Scales in Pediatric Orthopaedic Publications. *J Pediatr Orthop* 2019;**39**:e153–8. doi:10.1097/BPO.0000000000001263

13 Phillips L, Carsen S, Vasireddi A, *et al.* Use of Patient-reported Outcome Measures in Pediatric Orthopaedic Literature. *J Pediatr Orthop* 2018;**38**:393–7. doi:10.1097/BPO.0000000000000847

14 Black N. Patient reported outcome measures could help transform healthcare. *BMJ* 2013;**346**. doi:10.1136/bmj.f167

15 Kirkham JJ, Boers M, Tugwell P, *et al.* Outcome measures in rheumatoid arthritis randomised trials over the last 50 years. *Trials* 2013;**14**:324. doi:10.1186/1745-6215-14-324

For peer review only - http://bmjopen.bmj.com/site/about/guidelines.xhtml

16 Kirkham JJ, Clarke M, Williamson PR. A methodological approach for assessing the uptake of core outcome sets using ClinicalTrials.gov: findings from a review of randomised controlled trials of rheumatoid arthritis. *BMJ* 2017;:j2262. doi:10.1136/bmj.j2262

17 Williamson PR, Altman DG, Blazeby JM, *et al.* Developing core outcome sets for clinical trials: Issues to consider. *Trials* 2012;**13**. doi:10.1186/1745-6215-13-132

18 Williamson PR, Altman DG, Bagley H, *et al.* The COMET Handbook: version 1.0. *Trials* 2017;**18**:280. doi:10.1186/s13063-017-1978-4

19 Kirkham JJ, Davis K, Altman DG, *et al.* Core Outcome Set-STAndards for Development: The COS-STAD recommendations. *PLoS Med* 2017;**14**:e1002447. doi:10.1371/journal.pmed.1002447

20 Kirkham JJ, Gorst S, Altman DG, *et al.* Core Outcome Set-STAndards for Reporting: The COS-STAR Statement. *PLoS Med* 2016;**13**:e1002148. doi:10.1371/journal.pmed.1002148

21 Ollivere BJ, Marson BA, Haddad FS. Getting the right answer: core outcome sets in orthopaedics. *Bone Joint J* 2019;**101-B**:233–4. doi:10.1302/0301-620X.101B3.BJJ-2019-0065

22 Haywood KL, Griffin XL, Achten J, *et al.* Developing a core outcome set for hip fracture trials. *Bone Joint J* 2014;**96-B**:1016–23. doi:10.1302/0301-620X.96B8.33766

23 Dorman SL, Shelton JA, Stevenson RA, *et al.* Management of medial humeral epicondyle fractures in children: a structured review protocol for a systematic review of the literature and identification of a core outcome set using a Delphi survey. Trials. 2018;**19**:119. doi:10.1186/s13063-018-2472-3

24 Kirkham JJ, Gorst S, Altman DG, *et al.* Core Outcome Set-STAndardised Protocol Items: the COS-STAP Statement. *Trials* 2019;**20**:116. doi:10.1186/s13063-019-3230-x

25 Marson BA, Deshmukh SR, Grindlay D, *et al.* A systematic review of outcomes measured in trials of treatments for fractures in children. PROSPERO 2018: CRD42018106605. PROSPERO. 2018.http://www.crd.york.ac.uk/PROSPERO/display_record.php?ID=CRD42018106605 (accessed 3 Oct 2018).

26 Moher D, Liberati A, Tetzlaff J, *et al.* Preferred Reporting Items for Systematic Reviews and Meta-Analyses: The PRISMA Statement. *PLoS Med* 2009;**6**:e1000097. doi:10.1371/journal.pmed.1000097

27 WHO. International Classification of Functioning, Disability and Health (ICF). WHO. 2001.http://www.who.int/classifications/icf/en/ (accessed 5 Sep 2018).

28 Young AE, Davies A, Bland S, *et al.* Systematic review of clinical outcome reporting in randomised controlled trials of burn care. BMJ Open. 2019;**9**. doi:10.1136/bmjopen-2018-025135

29 A. M, N. B, J. M, *et al.* Toward a Core Outcome Set for Head, Neck, and Respiratory Disease in Mucopolysaccharidosis Type II: Systematic Literature Review and Assessment of Heterogeneity in Outcome Reporting. *J Inborn Errors Metab Screen* 2019;**7**. doi:10.1177/2326409818816721

30 Hancox JM, Toman E, Brace-McDonnell SJ, *et al.* Patient-centred outcomes for prehospital trauma trials: A systematic review and patient involvement exercise. *Trauma (United Kingdom)* Published Online First: 1 October 2019. doi:10.1177/1460408618817912

31 O'Reilly M, Dogra N. Planning your Interview: Key Decisions and Practical Issues. In: O'Reilly M, Dogra N, eds. *Interviewing Children and Young People for Research*. 1 Oliver's Yard, 55 City

For peer review only - http://bmjopen.bmj.com/site/about/guidelines.xhtml

Road London EC1Y 1SP: : SAGE Publications Ltd 2017. 71–94. doi:10.4135/9781526419439.n5

32 Bernheimer LP. The use of qualitative methodology in child health research. *Child Health Care* 1986;**14**:224–32. doi:10.1207/s15326888chc1404_6

33 Zajac R, Brown DA. Conducting Successful Memory Interviews with Children. *Child Adolesc Soc Work J* 2018;**35**:297–308. doi:10.1007/s10560-017-0527-z

34 Larsson AS, Lamb ME. Making the most of information-gathering interviews with children. *Infant Child Dev* 2009;**18**:1–16. doi:10.1002/icd.573

35 Morse JM. Determining Sample Size. *Qual Health Res* 2000;**10**:3–5. doi:10.1177/104973200129118183

36 Holloway I, Galvin K. Sampling Stratergies. In: Holloway I, Galvin K, eds. *Qualitative research in nursing and healthcare*. Hoboken: : John Wiley & Sons, Incorporated 2016. 141–56.

37 Selb M, Escorpizo R, Kostanjsek N, *et al.* A guide on how to develop an International Classification of Functioning, Disability and Health Core Set. *Eur J Phys Rehabil Med* 2015;**51**:105–17.

38 Manning JC, Hemingway P, Redsell SA. Stories of survival: Children's narratives of psychosocial well-being following paediatric critical illness or injury. *J Child Heal Care* 2017;**21**:236–52. doi:10.1177/1367493517717078

39 Coad J. Using art-based techniques in engaging children and young people in health care consultations and/or research. *J Res Nurs* 2007;**12**:487–97. doi:10.1177/1744987107081250

40 Cieza A, Fayed N, Bickenbach J, *et al.* Refinements of the ICF Linking Rules to strengthen their potential for establishing comparability of health information. *Disabil Rehabil* 2016;**8288**:1–10. doi:10.3109/09638288.2016.1145258

41 Cieza A, Geyh S, Chatterji S, *et al.* ICF linking rules: an update based on lessons learned. *J Rehabil Med* 2005;**37**:212–8. doi:10.1080/16501970510040263

42 Jones J, Hunter D. Consensus methods for medical and health services research. *BMJ* 1995;**311**:376–80. doi:10.1136/bmj.311.7001.376

43 Helmer-Hirschberg O. Analysis of the future: the Delphi Method. Santa Monica, CA: 1967.

44 Boulkedid R, Abdoul H, Loustau M, *et al.* Using and reporting the Delphi method for selecting healthcare quality indicators: a systematic review. *PLoS One* 2011;**6**:e20476. doi:10.1371/journal.pone.0020476

45 Sinha IP, Smyth RL, Williamson PR. Using the Delphi Technique to Determine Which Outcomes to Measure in Clinical Trials: Recommendations for the Future Based on a Systematic Review of Existing Studies. *PLoS Med* 2011;**8**:e1000393. doi:10.1371/journal.pmed.1000393

46 Gargon E, Crew R, Burnside G, *et al.* Higher number of items associated with significantly lower response rates in COS Delphi surveys. *J Clin Epidemiol* 2019;**108**:110–20. doi:10.1016/J.JCLINEPI.2018.12.010

47 MacLennan S, Kirkham J, Lam TBL, *et al.* A randomized trial comparing three Delphi feedback strategies found no evidence of a difference in a setting with high initial agreement. *J Clin Epidemiol* 2018;**93**:1–8. doi:10.1016/j.jclinepi.2017.09.024

48 Blazeby JM, Macefield R, Blencowe NS, *et al.* Core information set for oesophageal cancer

For peer review only - http://bmjopen.bmj.com/site/about/guidelines.xhtml

surgery. *Br J Surg* 2015;**102**:936–43. doi:10.1002/bjs.9840

49 Potter S, Holcombe C, Ward JA, *et al.* Development of a core outcome set for research and audit studies in reconstructive breast surgery. *Br J Surg* 2015;**102**:1360–71. doi:10.1002/bjs.9883

50 Harman NL, Bruce IA, Kirkham JJ, *et al.* The importance of integration of stakeholder views in core outcome set development: Otitis Media with Effusion in children with cleft palate. *PLoS One* 2015;**10**:1–22. doi:10.1371/journal.pone.0129514

51 Prinsen CAC, Vohra S, Rose MR, *et al.* How to select outcome measurement instruments for outcomes included in a " Core Outcome Set " – a practical guideline. *Trials* 2016;**17**. doi:10.1186/s13063-016-1555-2

Figure legends

Figure 1: Study flow chart for the three round Delphi study. Outcome domains will be identified as 'consensus in' if 70% of panellists rate the outcome domain with a score of 7-9 and fewer than 15% of panellists score the domain 1-3. Outcome domains will be identified as 'consensus out' if 70% of panellists score the outcome domain 1-3 and fewer than 15% of panellists score the domain 7-9.

Figure 2: Study flow for consensus meeting. Important outcomes will be identified through voting following each breakout session. Important outcomes will then be ranked and further voting will be completed to identify the outcomes for inclusion in the core outcome set

For peer review only - http://bmjopen.bmj.com/site/about/guidelines.xhtml

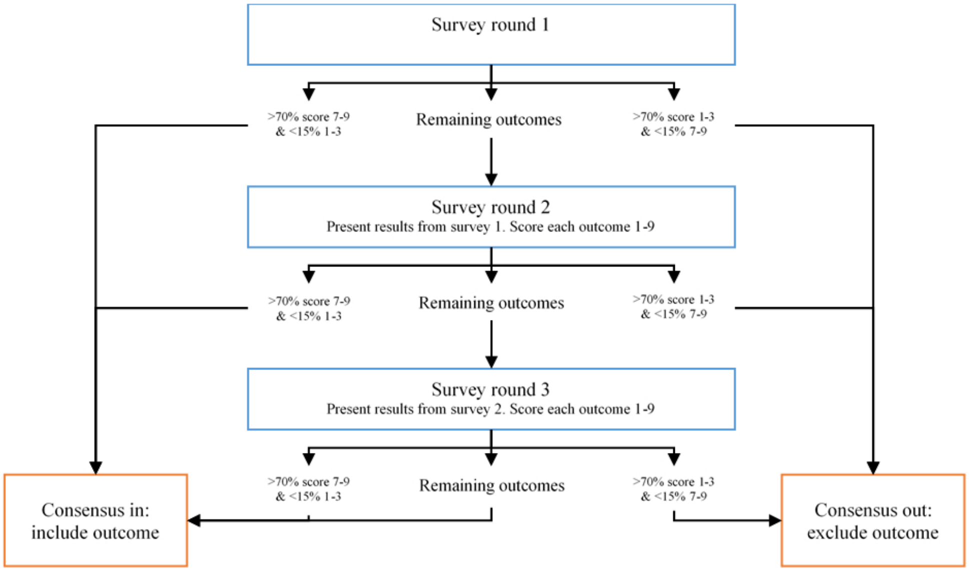

Study flow chart for the three round Delphi study. Outcome domains will be identified as 'consensus in' if 70% of panellists rate the outcome domain with a score of 7-9 and fewer than 15% of panellists score the domain 1-3. Outcome domains will be identified as 'consensus out' if 70% of panellists score the outcome domain 1-3 and fewer than 15% of panellists score the domain 7-9.

82x50mm (300 x 300 DPI)

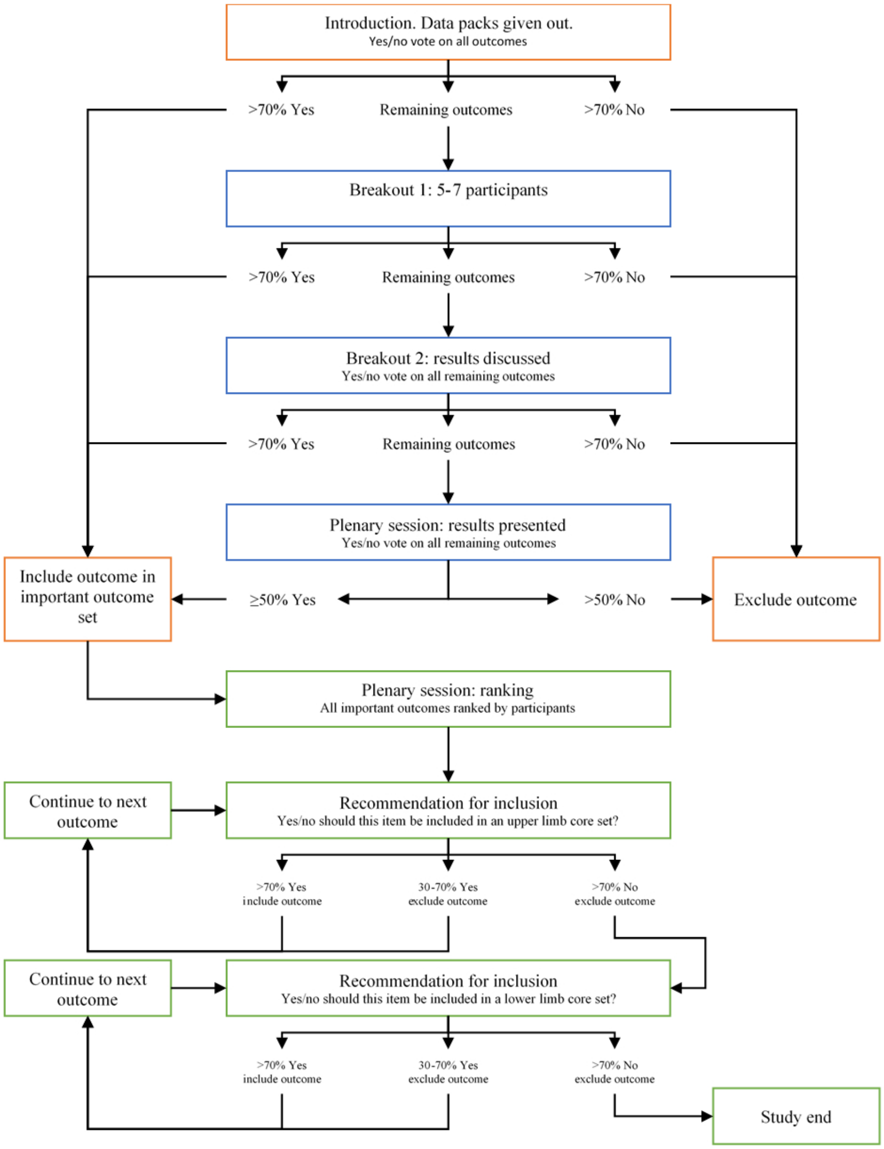

Figure 2 Study flow for consensus meeting. Important outcomes will be identified through voting following each breakout session. Important outcomes will then be ranked and further voting will be completed to identify the outcomes for inclusion in the core outcome set

82x107mm (300 x 300 DPI)

TITLE/ABSTRACT			Page
Title	1a	Identify in the title that the paper describes the protocol for the planned development of a COS	1
Abstract	1b	Provide a structured abstract	2
INTRODUCTION			
Background and objectives	2a	Describe the background and explain the rationale for developing the COS, and identify the reasons why a COS is needed and the potential barriers to its implementation	4
	2b	Describe the specific objectives with reference to developing a COS	4
Scope	3a	Describe the health condition(s) and population(s) that will be covered by the COS	5
	3b	Describe the intervention(s) that will be covered by the COS	5
	3c	Describe the context of use for which the COS is to be applied	5
METHODS			
Stakeholders	4	Describe the stakeholder groups to be involved in the COS development process, the nature of and rationale for their involvement and also how the individuals will be identified; this should cover involvement both as members of the research team and as participants in the study	5,8
Information sources	5a	Describe the information sources that will be used to identify the list of outcomes. Outline the methods or reference other protocols/papers	5
	5b	Describe how outcomes may be dropped/combined, with reasons	7
Consensus process	6	Describe the plans for how the consensus process will be undertaken	8
Consensus definition	7a	Describe the consensus definition	9
	7b	Describe the procedure for determining how outcomes will be added/combined/dropped from consideration during the consensus process	9

ANALYSIS			
Outcome scoring/feedback	8	Describe how outcomes will be scored and summarised, describe how participants will receive feedback during the consensus process	7-9
Missing data	9	Describe how missing data will be handled during the consensus process	na
ETHICS and DISSEMINATION			
Ethics approval/informed consent	10	Describe any plans for obtaining research ethics committee/institutional review board approval in relation to the consensus process and describe how informed consent will be obtained (if relevant)	10
Dissemination	11	Describe any plans to communicate the results to study participants and COS users, inclusive of methods and timing of dissemination	10
ADMINISTRATIVE INFORMATION			
Funders	12	Describe sources of funding, role of funders	10
Conflicts of interest	13	Describe any potential conflicts of interest within the study team and how they will be managed	10

only
