# Supplementary material for: CORE-Kids: a protocol for the development of a core outcome set for childhood fractures
Source: BMJ Open. 2020 Feb 28;10(2):e036224. doi: 10.1136/bmjopen-2019-036224 (PMC7050303; doi:10.1136/bmjopen-2019-036224)
Supplement: Reviewer comments [file bmjopen-2019-036224.reviewer_comments.pdf]

PEER REVIEW HISTORY

BMJ Open publishes all reviews undertaken for accepted manuscripts. Reviewers are asked to complete a checklist review form (**http://bmjopen.bmj.com/site/about/resources/checklist.pdf**) and are provided with free text boxes to elaborate on their assessment. These free text comments are reproduced below.

ARTICLE DETAILS

**TITLE (PROVISIONAL)**	CORE-Kids: A protocol for the development of a core outcome set for childhood fractures
**AUTHORS**	Marson, Ben; Manning, Joseph; James, Marilyn; Craxford, Simon; Deshmukh, Sandeep; Ollivere, Benjamin

This article was not externally reviewed at BMJ Open. Protocols that have been independently assessed prior to submission to BMJ Open are usually fast-tracked to publication on the grounds that further substantial changes will not be possible. This independent assessment will usually be external, independent review for both a substantial grant award from a non-commercial or government funder and ethics approval.
